# 3D Printable Polymer Electrolytes for Ionic Conduction based on Protic Ionic Liquids

**DOI:** 10.1002/cphc.202400849

**Published:** 2025-01-08

**Authors:** Alyna Lange, Sajal Arwish, Aurelie Rensonnet, Khalid Elamin, Iqbaal Abdurrokhman, Zaneta Wojnarowska, Mark Rosenwinkel, Cedric Malherbe, Monika Schönhoff, Kerstin Zehbe, Andreas Taubert

**Affiliations:** ^1^ Institute of Chemistry University of Potsdam Karl-Liebknecht-Straße 24–25 14476 Potsdam-Golm Germany; ^2^ Institute of Physical Chemistry University of Münster Corrensstraße 28/30 48149 Münster Germany; ^3^ Mass Spectrometry Laboratory University of Liege 11 Allee du 6 aout 4020 Liege Belgium; ^4^ Department of Chemistry and Chemical Engineering, Applied Chemistry Chalmers University of Technology Gothenborg 41296 Sweden; ^5^ Institute of Physics The University of Silesia in Katowice 75 Pułku Piechoty 1 A 41-500 Chorzow Poland

**Keywords:** Room temperature ionic liquids, Polymer electrolytes, Ion conduction, 3D printing, Stereolithography

## Abstract

A range of protic ionic liquids (PILs) based on tri‐n‐alkylammonium cations and mesylate/triflate anions were incorporated into a polymer matrix to form ionogels (IGs). These systems were investigated for their thermal and electrochemical behaviour, as well as under the aspect of ion motion via PFG‐NMR. The ionic conductivities of the ILs/IGs are in the range of 10^−4^–10^−3^ S/cm^−1^ at elevated temperatures and the diffusion coefficients are around 10^−11^ m^2^ s^−1^. Successful 3D printing of an IG with 70 wt % of IL is possible via stereolithography approaches, opening up applications in, e. g., structured ion‐conductive membranes.

## Graphical abstract

Protic ionic liquids and a suitable matrix yield soft, flexible and highly conductive materials with application potential in e.g. fuel cells. The materials can be printed using stereolithographic 3D printing and show a good thermal and chemical stability while mantaining good ion conduction at all temperatures investigated.

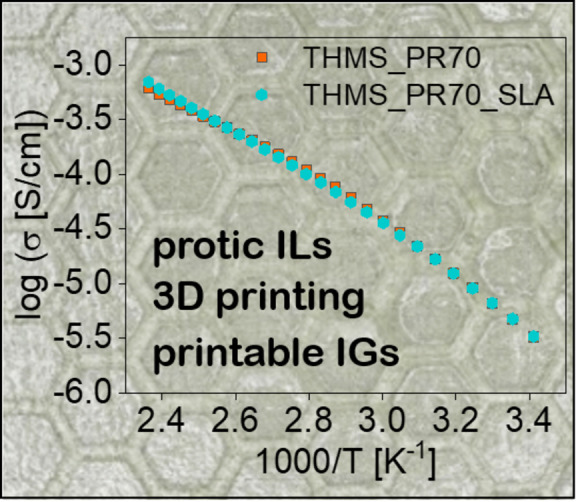

## Introduction

In light of the well‐known worldwide energy and environmental challenges, there is a constantly growing need for advanced and sustainable energy technologies. Along with this, the improvement of existing energy solutions is very much in the focus of current research and technology. Among others, batteries and fuel cells are key devices in future energy storage strategies. One of the main and often decisive components in these technologies is the electrolyte: advanced electrolytes should have high ionic conductivities, excellent chemical and thermal stabilities and large electrochemical windows. At the same time, these electrolytes should be cheap and electrolyte production should be flexible, adaptable, and sustainable.[^1^, ^2^, ^3^]

Many common electrolytes suffer from leaking or from safety issues. A solution to some of these problems is the immobilization of the electrolyte into a quasi‐solid state membrane. Among those, gel polymer electrolytes (GPEs) have become highly popular and are now widely investigated and used.^[4]^ GPEs combine the advantageous properties of both worlds–a solid polymer host and a liquid electrolyte guest. GPEs therefore offer mechanical stability, high ionic conductivity, reduced leakage, and improved safety.

When ionic liquids (ILs) are used as the solvent in an electrolyte, GPEs are commonly referred to as ionogels (IGs).[^5^, ^6^] ILs have long been investigated for their promising properties such as negligible vapor pressure, high ionic conductivities, non‐flammability, and wide electrochemical stability windows; all of this makes them interesting candidates as electrolytes. ILs are comprised solely of ions and there are countless possible combinations for anions and cations.[^7^, ^8^] Each combination provides ILs with different properties and therefore applications or application potential.

Protic ILs (PILs) are an IL sub‐class that can be obtained by Brønsted acid‐base reaction. PILs have found application in many fields, including organic synthesis, chromatography, mass spectrometry, or biochemistry and bioengineering.[^9^, ^10^] Owing to their acidic nature and the possibility of proton transfer and conduction, PILs are promising candidates as electrolytes for, e. g., polymer electrolyte membrane fuel cells (PEMFCs) or batteries among others.[^6^, ^11^, ^12^, ^13^, ^14^] Commonly, the membrane material of choice in PEMFCs is Nafion, a perfluorinated polymer with side chains functionalized with strongly acidic −SO_3_H groups.[^15^, ^16^, ^17^] While generally, the resulting fuel cells show a good performance, there are a few challenges that have yet to be resolved.

The most important issue is the rather low operating temperature of Nafion‐based membranes of only up to 80 °C. At higher temperatures, Nafion membranes show significant dehydration and failure of the respective fuel cell.[^11^, ^18^] As a result, alternative membrane materials are highly sought after; this especially applies to membrane materials with appropriate thermal and chemical stabilities, defined (pore or channel) geometries and sizes, high ion mobilities, and correspondingly high ionic conductivities at elevated temperatures. Given their general properties, PILs are prime candidates for application in PIL‐based membranes. However, as liquids are not suited for membrane formation, the PILs must be incorporated and immobilized in a suitable host matrix to produce suitable solidified liquid electrolytes.

In principle, there are three options to immobilize an IL and to prepare IL‐based polymer gels^[19]^: (1) swelling of polymers with the IL,[^18^, ^20^, ^21^] (2) polymerization of polymerizable ILs,[^22^, ^23^] and (3) curing of (vinyl) monomers in an IL.[^24^, ^25^] Indeed, we have previously shown that ionogels based on PILs can be manufactured and structured via a stereolithography (SLA) 3D printing approach.^[26]^ This method provides a first prototype of a 3D printed ion‐conductive electrolyte which can naturally be extended from fuel cells to e. g. batteries and beyond. Especially in the last couple of years, research into 3D printable IGs for various applications, but especially sensors, has gained increasing importance.[^27^, ^28^, ^29^, ^30^]

This is particularly interesting because SLA‐based approaches could alleviate one of the major problems in electrochemical devices: the non‐optimal contact of electrolyte and electrode.[^31^, ^32^, ^33^] If the electrode and the electrolyte are in stable and intimate contact by way of a properly designed SLA process, this highly important contact is naturally enhanced and a much better performance and better long‐term stability of the entire device can be expected.[^34^, ^35^] As a result, SLA is a promising approach for the design of three‐dimensional ionogels (IGs) to produce flexible and complex designs for membranes and on the longer run also for entire electrochemical devices with complex architectures.[^36^, ^37^, ^38^, ^39^]

**Scheme 1:**
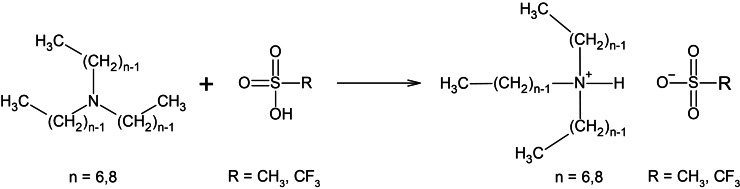
Protic ionic liquids by mixture of a sulfonic acid and tertiary amines.

The main goal of the current study therefore is to improve upon existing IL/resin combinations that are suitable for SLA printing for the production of stable and robust IGs with high conductivities. These IGs may have an application potential in e.g. fuel cells or battery membranes. The article therefore describes a set of PILs that are formed by neutralisation of methanesulfonic or trifluoromethanesulfonic acid and tertiary amines (Scheme 1). The protic IL tri‐n‐ethylammonium mesylate (TEMS) is well known for its low melting point and high ionic conductivity.[^21^, ^40^] Based on these properties this IL is an interesting candidate as the IL component in IGs and has already been used as such.[^21^, ^41^] Both, mesylate and triflate have also been used as anions in ILs. It has hereby already been shown that mesylate based ILs can act as a true proton conductor via Grotthus mechanism in contrast to ILs containing a more sterically hindered anion.^[42]^ As the triflate anion is known to improve the thermal properties and also conductivities compared to mesylate, this anion was chosen as comparison in our study.[^21^, ^43^, ^44^]

The resulting ILs were characterized by spectroscopic and electrochemical methods and investigated under aspects of ionic motion. In contrast to many other PILs, the PILs obtained here are liquid at temperatures as low as −75 °C and have a visibly lower viscosity at room temperature compared to PILs previously studied in our group.^[26]^ They therefore offer an excellent processability during SLA printing and IG fabrication. They also mix well with the commercial SLA resin to form flexible and transparent IGs with high conductivities of the final 3D printed membranes.

## Experimental

**Materials**. Methanesulfonic acid (99 %), trihexylamine (97 %), and trioctylamine (98 %) were obtained from Aldrich Chemical Co. Ferrocene (99 %) and trifluoromethanesulfonic acid (98 %) were purchased from Alfa Aesar, acetonitrile (99.9 %) was obtained from VWR. Tetrabutylammonium hexafluorophosphate (98 %) was obtained from Merck.

Printable clear elastic Photoreactive Resin Formlabs (methacrylated oligomers (≥75–≤90 %), methacrylated monomers (≥25–≤50 %), and photo initiator (≤1 %) Diphenyl (2,4,6‐trimethylbenzoyl)phospine oxide) was obtained from 3D dimensional Co. All chemicals and materials were used as received.

### Preparation of Trihexylammonium Mesylate (THMS)

In a 500 mL round‐bottom flask trihexylamine (148.27 g, 0.55 mol) was cooled with an ice‐bath and stirred with a magnetic stir bar. An equimolar amount of methanesulfonic acid (52.87 g, 0.55 mol) was slowly added via a syringe and the mixture was stirred at 40 °C for 3 hours. The resulting highly viscous product was dried in vacuo (10^−3^ mbar). ^1^H NMR (400 MHz, CDCl_3_): δ [ppm]: 0.67–0.85 (m, 9 H) 1.20 (m, 18 H) 1.54–1.73 (m, 6 H) 2.56–2.69 (m, 3 H) 2.81–3.00 (m, 6 H).

The reproducibility of this synthetic procedure and the homogeneity between different batches can be seen in NMR, DSC and CV data of different batches of THMS (Figures S1–S3).

### Preparation of Trioctylammonium Mesylate (TOMS)

In a 100 mL round‐bottom flask trioctylamine (14.17 g, 0.04 mol) was cooled with an ice‐bath and stirred with a magnetic stir bar. An equimolar amount of methanesulfonic acid (3.85 g, 0.04 mol) was slowly added via a syringe and the mixture was stirred at 40 °C for 3 hours. The resulting highly viscous product was dried in vacuo (10^−3^ mbar). ^1^H NMR (400 MHz, CDCl_3_): δ [ppm]: 0.64 (t, J=6.75 Hz, 9 H) 0.80–1.29 (m, 30 H) 1.43–1.59 (m, 6 H) 2.50 (s, 3 H) 2.69–2.91 (m, 6 H).

### Preparation of Trihexylammonium Triflate (THOTf)

In a 100 mL round‐bottom flask trihexylamine (7.22 g, 0.03 mol) was cooled with an ice‐bath and stirred with a magnetic stir bar under nitrogen. An equimolar amount of triflic acid (4.02 g, 0.03 mol) was slowly added via a syringe under a constant nitrogen flow and the mixture was stirred at 40 °C for 3 hours. The resulting viscous product was dried in vacuo (10^−3^ mbar). ^1^H NMR (400 MHz, CDCl_3_): δ [ppm]: 0.85 (br t, J=6.13 Hz, 9 H) 1.16–1.41 (m, 18 H) 1.57–1.80 (m, 6 H) 2.89–3.10 (m, 6 H).

### Preparation of Trioctylammonium Triflate (TOOTf)

In a 100 mL round‐bottom flask trioctylamine (11.97 g, 0.03 mol) was cooled with an ice‐bath and stirred with a magnetic stir bar under nitrogen. An equimolar amount of triflic acid (5.09 g, 0.03 mol) was slowly added via a syringe under a constant nitrogen flow and the mixture was stirred at 40 °C for 3 hours. The resulting viscous product was dried in vacuo (10^−3^ mbar). 1H NMR (400 MHz, CDCl_3_): δ [ppm]: 0.76–0.95 (m, 9 H) 1.14–1.42 (m, 30 H) 1.58–1.77 (m, 6 H) 2.90–3.15 (m, 6 H).

### Ionogel Preparation & Nomenclature

In all cases 5 g of IG were prepared by mixing 1.5 g of the photoreactive resin with 3.5 g of the respective IL with a speed mixer (3500 rpm, 90 s). The mixture was then cured in a Formlabs Form Cure instrument UV chamber at 405 nm for 45 min, at room temperature. IG nomenclature is as follows: IL_PR70; IL indicates the type of IL used for IG fabrication, PR is printable photoreactive resin, and 70 is the amount of IL in wt %. For example: TOMS_PR70 is an ionogel made of 70 wt % of TOMS and 30 wt % of resin. THMS_PR70_SLA indicates the 3D printed version of the respective IG.

### Ionogel Preparation for NMR‐diffusion Measurements

For NMR spectroscopy experiments, the IGs were prepared as described above by mixing appropriate amounts of IL and photoreactive resin with a speed mixer. While still liquid, the mixture of IL and resin was then directly filled into NMR tubes and cured in the UV chamber. After curing the NMR tubes were sealed with standard caps.

### Stereolithography (SLA, 3D Printing)

3D printing of the IGs was done on a Form 2 instrument from 3D dimensional Co. using 70 wt % of IL. For filling of the LT resin tank (Form 2) 158 g of THMS were mixed with 68 g of photoreactive resin (elastic clear Form 2) with a speed mixer (3500 rpm) for 90 seconds. A 3D model of the print was obtained from https://www.thingiverse.com/thing: 2616514. The file was directly uploaded to the 3D printing machine Form 2 for the final print with a layer thickness of 0.025 mm. The final 3D print was post‐treated with UV light (λ=405 nm) for 45 min with a Form Cure instrument.

### Characterization and Instrumentation

**Nuclear magnetic resonance spectroscopy**. ^1^H‐NMR spectra were recorded on a Bruker AVANCE NEO 400 MHz Spectrometer in CDCl_3_ at room temperature.

**Pulsed Field Gradient (PFG) NMR Diffusion**. NMR diffusion measurements were conducted on a Bruker Avance 400 MHz NMR spectrometer equipped with gradient amplifiers, using either a gradient probe head with selective radiofrequency inserts for ^1^H or ^19^F, respectively, (Bruker Diff30, max. gradient strength 12 T/m) or a broad band diffusion probe head (Bruker, DIFF BB, max. gradient strength 17 T/m). The temperature was controlled by an air stream and calibrated with a Pt100 thermocouple (Greisinger electronics). The self‐diffusion coefficients were measured by PFG‐NMR using a stimulated echo pulse sequence and taking a series of spectra, varying the gradient strength, *g*. The diffusion coefficient for a distinct resonance was obtained from the decay of the signal intensity *I* with *g* according to the Stejskal‐Tanner equation:^[45]^

(1) $$\;I=I_{0}\exp\left(-\gamma^{2}\delta^{2}g^{2}D\left(\Delta -\frac{\delta }{3}\right)\right),$$

with the gyromagnetic ratio *γ* of the observed nucleus. An observation time *Δ* of 100 ms was used, and the gradient pulse length δ was set to 3 ms. All decays could be well fitted by the exponential function of Equation (1).

**Infrared spectroscopy**. IR spectra were recorded using the attenuated total reflection (ATR) mode on a Thermo Scientific NICOLET iS5 with ID7 ATR diamond probe head. Spectra were taken from 500 to 4000 cm^−1^ with a resolution of 2 cm^−1^ and 64 scans per measurement.

**Raman Spectroscopy**. Raman spectra were obtained directly in the ionic liquid (dried under vacuum for 48 h and kept under argon atmosphere) using a Labram 300 Raman spectrometer (Horiba) interfaced with a 532 nm DPSS laser (input power on the sample 40 mW) and with a 90° angle objective (×20). The spectra were recorded in the Raman wavenumber shift ranging from 200–1700 cm^−1^ or 200–3600 cm^−1^. The spectra were baseline corrected with a fourth polynomial order. For the PILs characterisation, the normalisation in the 200–1700 cm^−1^ region (low wavenumber region, LWR) was performed according to the ~1035 cm^−1^ band intensity, and according to ~2935 cm^−1^ band intensity for the 2700–3100 cm^−1^ region (high wavenumber region, HWR).

**Density measurements**. The partial density in of the sample was determined in a density oscillation tube (DMA 5000 M, Anton Paar, Graz).

**Thermal analysis**. Simultaneous thermogravimetric analysis‐differential thermal analysis (TGA‐DTA) experiments were done on a Mettler Toledo TGA/DSC 3+ Thermogravimetric Analyzer from 30 to 600 °C with a heating rate of 10 K/min in air in aluminium oxide crucibles. Differential scanning calorimetry (DSC) measurements were done on a Netzsch Polyma DSC 214. DSC traces were recorded from −100 to 150 °C using liquid nitrogen cooling and a heating rate of 10 °C/min. Isothermal times were 10 min. Samples of 10 mg were placed in aluminium pans with pierced lids. Heating and cooling cycles were repeated three times for reproducibility.

**Speedmixer**. A DAC 150 FVZ (Flacktek Inc.) speedmixer with a rotation speed of 3500 rpm and a mixing time of 90 s was used for mixing of the ionogel components prior to curing.

**Electrochemistry**. (CV) measurements were performed using the three‐electrode system of a Metrohm Autolab PGSTAT204 and the TSC 70 closed measuring cell by rhd instruments. The CV cell consisted of platinum wires as working (A=4.9×10^−4^ cm^2^) and reference electrode and a platinum crucible as counter electrode. Before measuring the ILC compounds were dried in‐vacuo (10^−2^ mbar) overnight. Afterwards the samples were solved in dry acetonitrile (0.05 M) and the solution deoxygenated by bubbling nitrogen through the solution. Ferrocene was utilized as internal standard (5×10^−4^ M) and tetra‐n‐butylammonium hexafluorophosphate (Bu_4_NPF_6_) as electrolyte (0.1 M). A scan rate of 0.1 V/s and three subsequent scans were used for all measurements. The ferrocene half wave potentials were normalized to a half wave potential of 0.0 V. For the analysis of the data the second scan of the measurements and a cut‐off current of 5×10^−7^ A was used.

**Dielectric spectroscopy (DS)**. Dielectric measurements were performed on a Novocontrol GmbH Alpha dielectric spectrometer covering the frequency and temperature ranges 10^−1^–10^7^ Hz and −100–150 °C, respectively. The value of the ionic conductivity was taken from the plateau region of the real part of each ionic conductivity spectrum. Stainless steel electrodes (diameter=15 mm) with a fixed distance provided by a quartz ring were used for the studies of the ILs and IGs. During the measurements, the temperature was controlled by a Novocool system using a nitrogen gas cryostat with an accuracy of 0.1 K. All samples were dried before measurements (60 °C, 2 h, vacuum).

## Results and Discussion

All ILs investigated in this study can be synthesized on a gram scale by an acid base reaction. Depending on the nature of the anion or the length of the alkyl chains on the cation the ILs show differences in properties such as thermal and electrochemical behaviour, Table 1. TEMS and TBMS show ionic conductivities in the range of 10^−2^–10^−3^ S/cm^−1^ at elevated temperatures,^[44]^ whereas the ionic conductivities for THMS and TOMS are slightly lower.

**Table 1:** Thermal data and ionic conductivities for the protic ionic liquids (PILs).

Ionic liquid [base]/[acid]=1/1	T_m_ [°C]^[a]^	T_g_ [°C]^[b]^	$\sigma_{120^{\circ }C}$ [S/cm]	Compatibility with SLA resin
**TEMS^[d]^**	17.4^[c]^	−62.1^[c]^	1.6×10^−2[c]^	limited
**TBMS^[d]^**	41.0^[c]^	−69.4^[c]^	7.5×10^−3[c]^	limited
**THMS**	‐	−75.3	7.5×10^−4^	good
**TOMS**	‐	−77.8	4.6×10^−4^	good
**THOTf**	−5.4	−83.6	1.2×10^−3^	limited
**TOOTf**	21.8	−62.3	5.0×10^−4^	poor

[a] Melting point, [b] Glass transition temperature, [c] Data taken from ref.^[44]^ σ_120 °C_: ionic conductivity at 120 °C, [d] ILs were not chosen for preparation of IGs, as they are solids at room temperature. Please note that there are different T_m_ for TEMS in the literature.[^10^, ^40^, ^44^, ^46^]

In contrast to TEMS and TBMS,[^40^, ^44^, ^46^] THMS and TOMS are liquid at room temperature. They therefore display a better compatibility with the photoreactive resin used to form IGs (see below) and the THMS/resin and TOMS/resin mixtures are easier to handle during IG fabrication than mixtures of the resin with TEMS and TBMS. For the largest part of the article, we will therefore focus on THMS and TOMS for IG preparation and analysis.

For a comparison of individual aspects of the ILs this study also includes experiments with ILs based on trihexyl‐ and trioctylamine with trifluoromethanesulfonic acid (triflic acid) rather than methane sulfonic acid. Generally, triflate based ILs like THOTf and TOOTf exhibit higher thermal stabilities and higher ionic conductivities when compared to their mesylate based counterparts.[^43^, ^47^] However, THOTf and TOOTf exhibit problems in terms of their miscibility with the SLA resin, similar to TEMS and TBMS. Especially TOOTf shows a quite limited compatibility with the photoreactive resin resulting in white, phase separated IGs in contrast to the transparent THMS_PR70 and TOMS_PR70 (see Figure S4). The article will therefore mainly focus on IGs made from THMS and TOMS and the IGs made from these two ILs. TEMS, TBMS, THOTf, and TOOTf along with the corresponding IGs will be used as a comparison to illustrate certain aspects.

**Figure 1:**
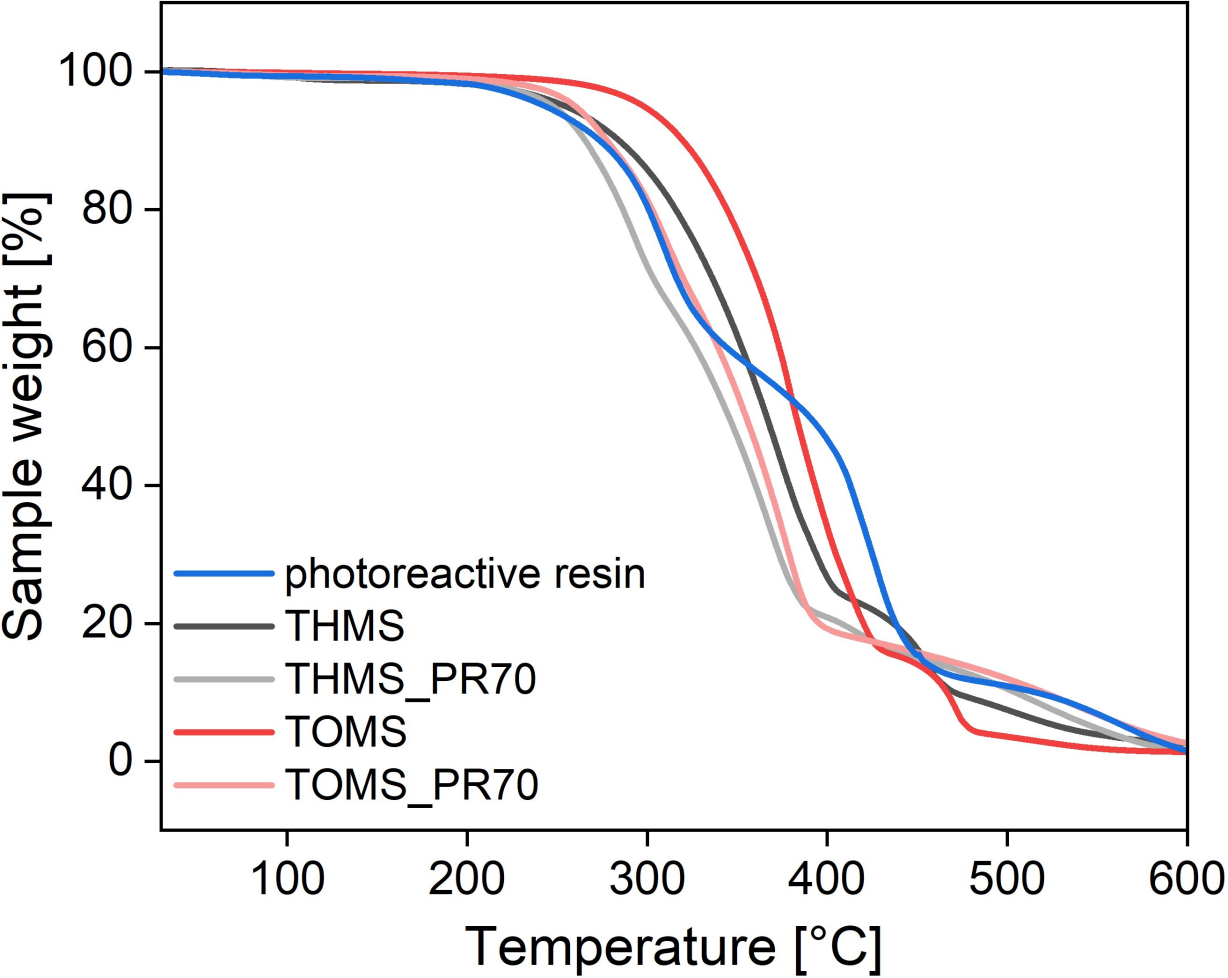
TGA curves of the mesylate based ILs, the pure photoreactive resin, and the respective ionogels.

Phase transitions and thermal stabilities of the ILs were investigated with differential scanning calorimetry (DSC) and thermogravimetric analysis (TGA), respectively. Figures 1, S5 and S6 show the respective TGA curves and Table 2 summarizes the TGA results of all compounds.

**Table 2:** TGA data of the photoreactive resin, the pure ILs THMS and TOMS, and the corresponding IGs.^[a]^

Thermal behavior			
Compound	T_150_ [%]	T_600_ [%]	T_5 %_ [°C]
**Photoreactive resin**	1.0	98.5	242.4
**THMS**	1.3	97.4	254.4
**THMS_PR70**	1.0	98.7	247.6
**TOMS**	0.3	98.5	297.5
**TOMS_PR70**	0.6	97.5	259.7
**THOTf**	0.3	92.0	340.2
**THOTf_PR70**	0.6	98.0	236.8
**TOOTf**	0.3	91.4	340.7
**TOOTf_PR70**	0.7	98.3	248.3

[a] T_5 %_: onset temperature of decomposition at 5 % weight loss, T_600_: weight loss at 600 °C, T_150_: weight loss at 150 °C.

THMS and TOMS decompose in a three step process. The first mass loss observed for THMS is 1.1 % up to 150 °C and indicates a loss of residual water. In contrast, TOMS shows a smaller mass loss up to 150 °C of only 0.3 %, which is in good agreement to its lower hygroscopy; this is likely due to the longer alkyl chains in TOMS. A second mass loss for both ILs, indicating the degradation of the alkyl chains, can be seen between 255–405 °C for THMS (−70.6 %) and 298–430 °C for TOMS (−78.7 %). The decomposition onset temperatures T5 % (at a mass loss of 5 %)[^10^, ^48^] are at 254.4 °C and 297.5 °C, respectively. These results agree with the trend shown by Nakamoto et al. where TBMS shows a higher thermal stability than TEMS and THMS shows a higher stability than TBMS.^[44]^ A third distinct decomposition step can be observed between 410–470 °C for THMS (−4.4 %) and between 430–482 °C for TOMS (−11.9 %). Complete decomposition of the ILs is observed until 600 °C.

The pure photoreactive resin shows a decomposition onset temperature T5 % at 242.4 °C, although DTA data suggest a first process starting around 170 °C, which could be associated with the loss of residual solvent or monomers. The decomposition of the resin takes place over three steps. The first step shows a mass loss of 41.7 % up to 375 °C. The second mass loss of 41.0 % is observed up to 470 °C. The last step shows a mass loss of 10.7 %.

Figure 1 also shows the TGA graphs for the IGs made from THMS and TOMS, respectively, using the SLA resin. The TGA data obtained from the IGs much more closely resemble the data obtained for the pure ILs than those obtained for the pure resin. The T5 % of THMS_PR70 and TOMS_PR70 are 247.6 °C and 259.7 °C. In agreement with the data of the pure ILs a mass loss of 1.1 % and 0.6 % is visible until 150 °C. The biggest mass losses of 73.9 % and 75.8 % can be seen between the respective T5 % and around 400 °C, respectively, with nearly complete decomposition reached at 600 °C.

When comparing the T_5 %_ of the pure ILs THMS and TOMS and their respective IGs a slightly lower thermal stability for the IGs is apparent. The incorporation of the ILs into the polymer matrix seems to decrease the onset of thermal decomposition of up to 10 °C in the case of THMS and almost 40 °C for TOMS. The reason for this is the lower thermal stability of the pure polymer resin. Overall, the ILs and IGs show thermal stabilities and a possible operation temperature window up to 170 °C.

THOTf and TOOTf, that is the triflate analogues of THMS and TOMS, exhibit a very similar decomposition behavior, as both show a T_5 %_ around 340 °C, which is 80 K and 40 K more stable than THMS and TOMS, see Table 2 and Figure S5. This observation is consistent with literature, as ILs based on the triflate anion are typically more stable towards thermal decomposition than ILs based on mesylate; the T_5 %_ of thermal decomposition in the triflate‐based ILs can be up to 100 °C higher than in mesylate‐based ILs.[^10^, ^43^, ^47^, ^49^]

The IGs based on the fluorinated ILs THOOTf and TOOTf, however, are overall slightly less stable than those based on THMS and TOMS as opposed to the thermal stability of the pure ILs. This might be caused by limited miscibilities of THOOTf and TOOTf with the SLA resin leading to phase separation in the resulting IGs (see Figure S4). This phase separation might lead to an initial degradation of the resin rich component at about 240 °C followed by a second decomposition step of the IL rich component at higher temperatures (see Figure S5).

The thermal behavior of the ILs and IGs was further investigated by differential scanning calorimetry (DSC). The pure photoreactive resin shows a poorly visible glass transition at T_g_=−73.2 °C and a very small endothermic peak at T_m_=19.3 °C upon heating (Figure S7). Due to the fact that the chemical nature of the resin is not exactly known it is difficult to assign this signal to a specific process.

**Figure 2:**
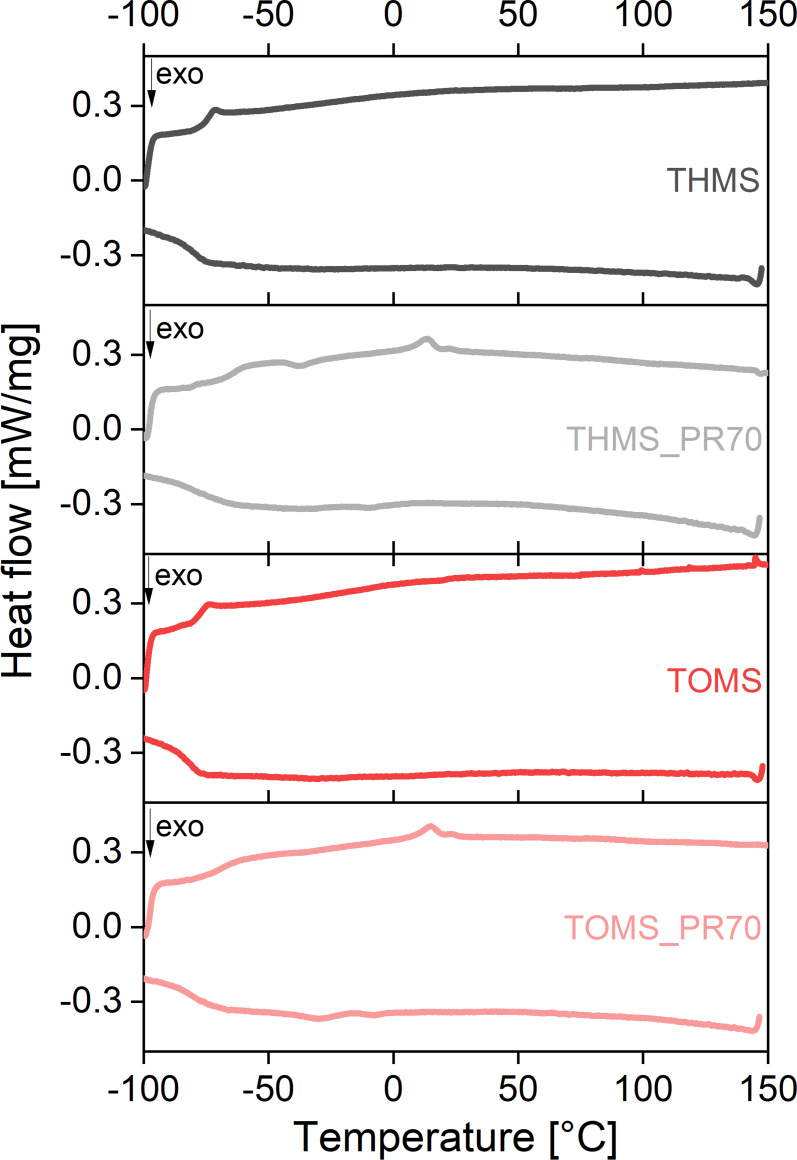
DSC heating and cooling traces (2^nd^ run) of mesylate based ILs and IGs.

Figure 2 shows the thermograms of the second heating and cooling cycle for THMS and TOMS and the corresponding IGs THMS_PR70 and TOMS_PR70, respectively. The DSC data for the pure ILs are almost identical with T_g_=−75.3 °C for THMS and T_g_=−77.8 °C for TOMS. The T_g_s are reproducible and can also be observed in the cooling curves. No (cold) crystallization or melting is visible in the investigated temperature range. This suggests a thermal application window for the ILs between −75 °C and +150 °C.

The IGs also show a similar behavior in the DSC. For both IGs, a T_g_ is observed at −64.1 °C (THMS_PR70) and −69.4 °C (TOMS_PR70) respectively. Both T_g_s are probably an overlap between the T_g_s of IL and pure resin. They show a temperature shift to higher T_g_s by about 10 °C compared to the pure ILs. These shifts hint at an influence of the polymer matrix on the dynamics of the ILs which seem to be slightly more hindered by the incorporation into the matrix. This phenomenon has also previously been shown in the literature.[^6^, ^50^, ^51^]

In contrast to the pure ILs, which do not show crystallization, the DSC traces of THMS_PR70 exhibit a very weak exothermic signal at T_c_=−43.5 °C and a subsequent very weak endothermic signal at T_m_=7.1 °C. These signals could be associated with a cold crystallization and corresponding melting process of a fraction of the IGs; possibly polymer side chain crystallization and melting process may cause this signal. For TOMS_PR70 the same endothermic process is observed at T_m_=6.9 °C,ek; but no corresponding exothermic signal is visible. Table 3 summarizes the temperatures and processes identified in DSC.

**Table 3:** Data of 2^nd^ DSC heating run of the investigated compounds.^[a]^

Compound	Thermal behavior		
Photoreactive resin	T_g_ [°C]	T_c_ [°C]	T_m_ [°C]
	−73.2	‐	19.3
**THMS**	−75.3	‐	‐
**THMS_PR70**	−64.1	−43.5	7.1
**TOMS**	−77.8	‐	‐
**TOMS_PR70**	−69.4	‐	6.9
**THOTf**	−83.6	−45.5	−5.4
**THOTf_PR70**	−76.6	‐	2.2
**TOOTf**	−62.3	‐	21.8
**TOOTf_PR70**	‐	‐	13.7

[a] T_c_=Cold crystallization.

Figure S8 shows the DSC traces for all fluorinated compounds. In contrast to THMS and TOMS, the fluorinated ILs THOTf and TOOTf show pronounced crystallization and melting processes in addition to glass transitions. The differences become even more pronounced once these ILs are incorporated into the resin to form IGs. THOTf_PR70 shows a glass transition and weak endothermic signal upon heating (comparable to THMS_PR70) with T_g_ being slightly higher than for its parent IL. However, TOOTf_PR70 shows a distinct and sharp melting peak, which can be assigned to the melting of TOOTf. This observation further supports the notion that this IG is a phase‐separated system with IL‐rich and resin‐rich regions. This phase separation has two consequences: 1. the IGs become turbid, and 2. each individual phase separated phase (matrix vs. IL rich phase) has its own thermal properties. This is why we observe an IL melting transition in the respective IG TOOTf_PR70.

**Figure 3:**
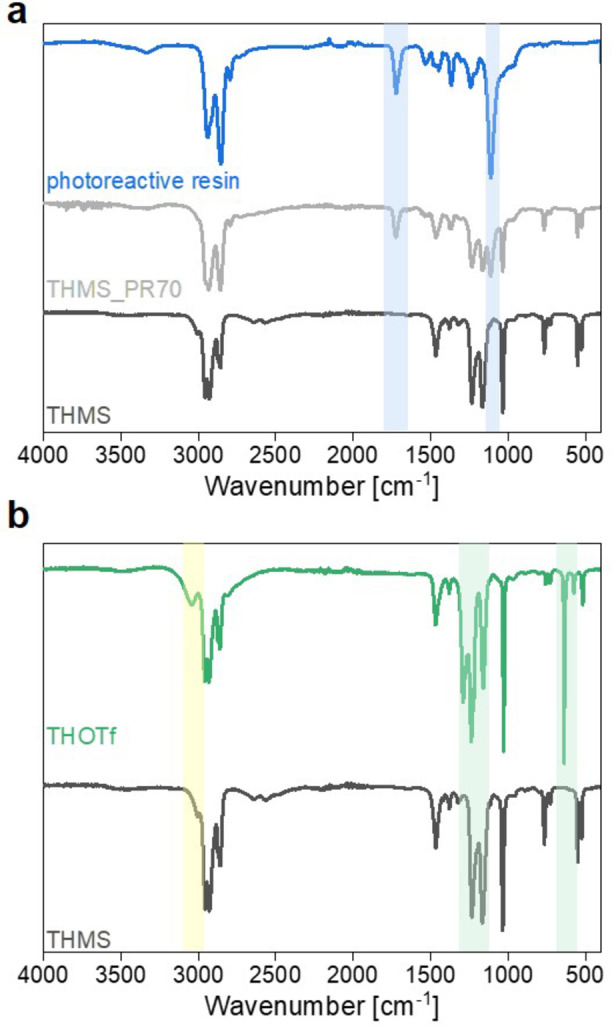
IR spectra of a) the photoreactive resin, the pure IL THMS and THMS_PR70 and b) the ILs THMS and THOTf (blue and green boxes to indicate differences, yellow box to highlight N−H stretching modes).

Attenuated total reflection infrared (ATR‐IR) spectra of the pure mesylate‐based ILs show typical bands associated with compounds based on alkylammonium and mesylate/triflate, see Figure 3. Strong bands between 3000–2850 cm^−1^ originate from aliphatic C−H‐stretching vibrations of the alkyl‐chains in the cation component of the ILs. In the pure ILs the weak band around 3006 cm^−1^ (yellow box in Figure 3b) can be attributed to “free” or poorly‐interacting N−H stretching modes, whereas in the range of 2800–2400 cm^−1^ a superposition of broad signals can be observed, which stems from “bonded” proton interaction with its surroundings, hinting at ion pairs. Both N−H modes are consistent with a proton transfer between a sulfonic acid and an amine and the concurrent formation of an ammonium cation.[^46^, ^52^, ^53^] A more detailed description and assignment of IL bands can be found in the SI (Section 4).

The IR spectra of the IGs show additional bands around 3430, 1540, 1110 cm^−1^, and more pronounced bands at 1730 and 1370 cm^−1^ (blue boxes in Figure 3a). These can be attributed to vibrations typical to the resin for which (based on IR spectroscopy) we tentatively infer a urethane‐methacrylate nature. As for the ILs a more detailed analysis can be found in Section 4 of the SI.

When comparing the IR spectra of the fluorinated ILs (Figures 3b and S9), with the ones of the mesylate based ILs additional bands can be seen that originate in the triflate anion (green boxes in Figure 3b). Spectra of the fingerprint region of THMS and THOTf for better comparison is shown in Figure S10. The strong band around 756 cm^−1^ (symmetric CF_3_ bending) and band around 574 cm^−1^ (asymmetric CF_3_ bending) can be attributed to free triflate anions.[^10^, ^54^, ^55^] IR spectra for all ILs and IGs can be found in Figure S9.

**Figure 4:**
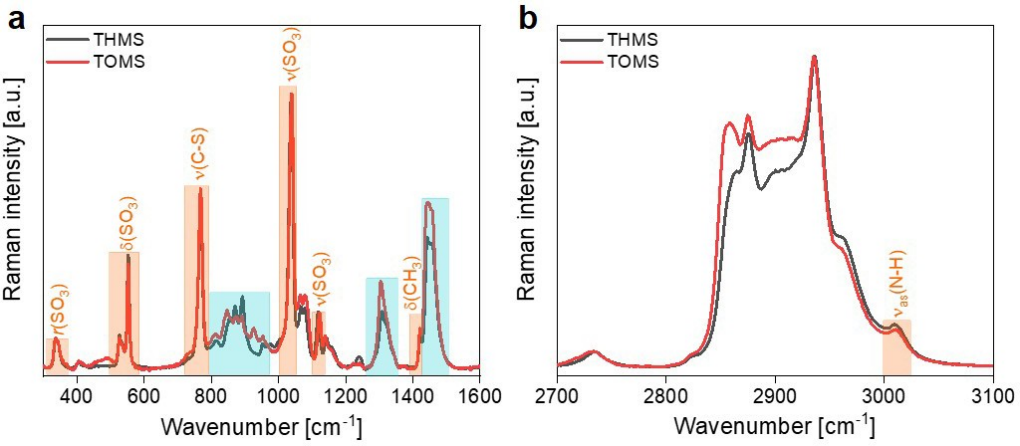
Raman spectra for THMS and TOMS in a) the low wavenumber region, and b) the high wavenumber region. (Blue boxes highlight cation bands and orange boxes highlight anion bands.)

In addition to IR spectroscopy, the PILs based on the weaker acid (methanesulfonic acid) were investigated via Raman spectroscopy. Figure 4 shows the respective spectra for THMS and TOMS in the low wavenumber and high wavenumber regions. The comparison in the low wavenumber region of the spectra of the two PILs shows an increase in intensity in TOMS of the bands related to the alkyl chain of the cation (the spectra being normalized according to the SO_3_ stretching bands attributed to the anion). This is in agreement with the higher number of CH_2_ groups in the tri‐octyl chains. In the high wavenumber region, the same observation applies, since the spectra are normalized according to the CH_3_ stretching band at 2938 cm^−1^. The low wavenumber region presents several typical bands of the mesylate anion, and the spectra in both regions are particularly different from the spectrum of pure methanesulfonic acid, as shown in Figure S11 that compares the spectra of THMS and the acid. The proton transfer between the amine and the acid, leading to alkylammonium cations coupled with mesylate anions, is demonstrated by the absence of the Raman signature related to the sulfonic OH group in the Raman spectrum of the IL (at 897 and 1164 cm^−1^). All band assignments for the Raman spectra can be found in Tables S1 and S2.

The electrochemical behavior of the ILs was studied via cyclic voltammetry (CV) as the ILs are intended for application in electrochemical devices. The electrochemical stability window (ΔEW=E_anodic_−E_cathodic_) is generally defined as the region in the voltammogram where no noteworthy Faradaic current is observed.[^56^, ^57^, ^58^] ILs based on the triflate anion usually show wider ΔEW as the triflate anion exhibits a higher stability towards oxidation, caused by the stronger delocalization of the negative charge.[^10^, ^47^, ^59^]

**Figure 5:**
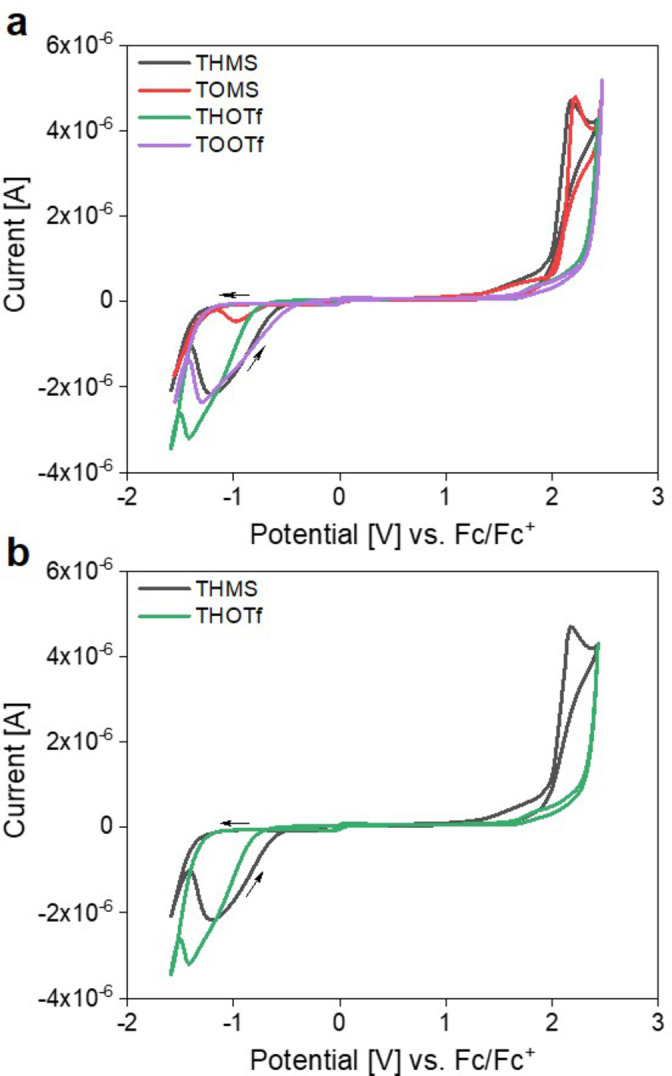
Cyclic voltammograms for a) all four PILs and b) THMS and THOTf only to enable a better comparison.

Figure 5a shows the voltammograms for THMS, TOMS, THOTf, and TOOTf for a direct comparison, and Figure 5b shows the cyclic voltammograms for THMS and THOTf for a better view of the data. It can clearly be seen that the oxidation process in THMS starts at lower anodic potentials when compared to THOTf. Therefore, the trend described above was confirmed in this study. Moreover, the oxidative process for the mesylate containing ILs exhibits a real oxidation peak, whereas the triflate‐based compounds only show an increasing current in the investigated window (Figure 5a). As +2.0 V is also the anodic potential limit for the solvent, potential current peaks or further oxidative processes for the triflate‐based ILs could not be studied in the chosen CV set‐up.

As a result, the oxidative limit for THOTf and TOOTf is ≥+2.0 V vs. Fc/Fc^+^. Whereas the anion nature seemingly influences the oxidative behavior, the alkyl chain length in the cation does not have a significant influence, Figures 5a and S12. When comparing the respective ILs (THMS vs. TOMS, THOTf vs. TOOTf) no significant difference in regards to peak maximum or cut‐off potential can be found.

For all ILs a similar behavior towards reduction is observed. While the mesylate ILs show a slightly higher stability, the cathodic cut‐off potentials are very close to each other overall.^[49]^ This cathodic process around −1.4 V can be attributed to the reduction of the ammonium‐cation, i. e., the reduction of protons.[^49^, ^54^, ^59^] At this point the importance of deoxygenation has to be stressed, as otherwise oxygen reduction as described by Khan et al. could be observed at negative potentials.^[60]^ Overall, the ILs show electrochemical stability windows between 3.1 and 3.4 V, similar to other published data,^[10]^ with the electrochemically most stable IL being TOOTf (Table 4).

**Table 4:** CV data of pure ILs and solvent.

Compound	E_an_ [V vs. Fc/Fc^+^]		E_cat_ [V vs. Fc/Fc^+^]	ΔEW_peak_ [V]	ΔEW_cut‐off_ [V]
	peak maximum	cut‐off	cut‐off		
**ACN**	‐	2.04	−2.46	‐	4.50
**THMS**	2.18	1.69	−1.40	3.58	3.09
**TOMS**	2.22	1.84	−1.36	3.58	3.20
**THOTf**	‐	2.01	−1.34	‐	3.35
**TOOTf**	‐	2.04	−1.34	‐	3.38

**Figure 6:**
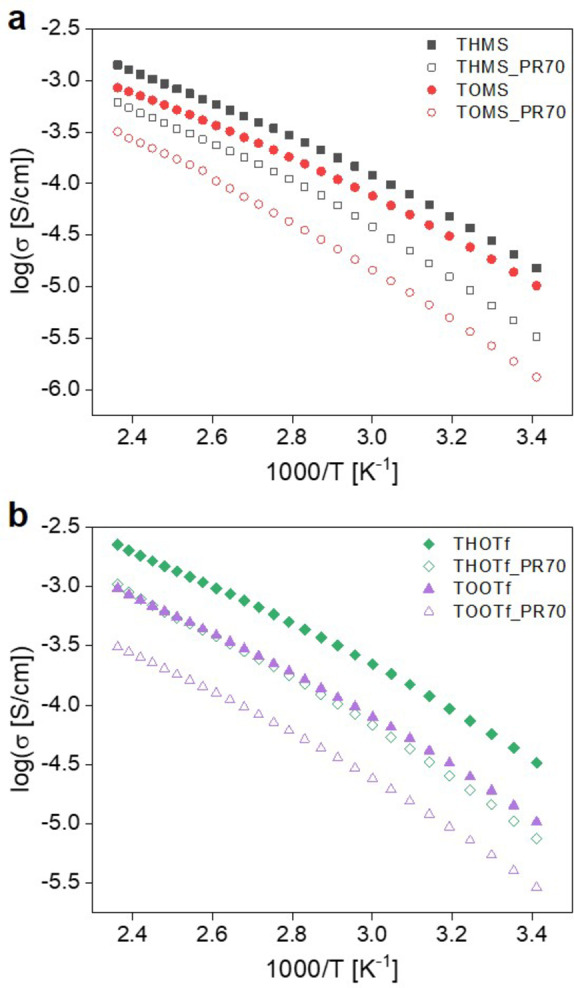
Conductivity data for a) mesylate based and b) triflate based ILs and the corresponding IGs. All materials were thoroughly dried before the measurements to eliminate water contributions to the conductivity as much as possible (see experimental section for details).

Ionic conductivities of the ILs and IGs were determined with broadband dielectric spectroscopy (BDS). As shown in Table 1, promising ionic conductivities for all four ILs (THMS, TOMS, THOTf and TOOTf) were obtained at elevated temperatures. For all ILs and their respective IGs measurements up to 150 °C were carried out, as this is a temperature of interest for fuel cell applications[^61^, ^62^] and also reflects the temperature window of the DSC measurements, up to which no irregularities or decomposition processes were found. Figures 6, S13 and Table 5 show the temperature dependent conductivities of the pure ILs and their IGs in the temperature range from 20 °C to 150 °C. The value of the ionic conductivity has been taken from the plateau region of each σ’(f) BDS spectrum. All ILs and IGs show higher conductivities at higher temperatures. It can be observed that a temperature difference of 130 °C already increases the conductivity by two orders of magnitude.^[49]^ THOTf shows the highest conductivities, followed by THMS, then TOOTf, and finally TOMS.

**Table 5:** IL and IG conductivities at RT and 150 °C.

Compound	$\sigma_{20^{\circ }C}$ [S/cm]	$\sigma_{150^{\circ }C}$ [S/cm]
**THMS**	1.5×10^−5^	1.8×10^−3^
**THMS_PR70**	3.3×10^−6^	6.1×10^−4^
**TOMS**	1.0×10^−5^	8.5×10^−4^
**TOMS_PR70**	1.3×10^−6^	3.2×10^−4^
**THOTf**	3.3×10^−5^	2.3×10^−3^
**THOTf_PR70**	5.8×10^−6^	1.0×10^−3^
**TOOTf**	1.0×10^−5^	9.6×10^−4^
**TOOTf_PR70**	2.9×10^−6^	3.1×10^−4^

Overall, the mesylate‐based ILs exhibit slightly lower conductivities when compared to the ILs with the triflate anion; the difference is more pronounced for the ILs with hexyl chains (see Figure S13a). The slightly higher ionic conductivities of the fluorinated compounds are in good agreement with results reported for other protic ionic liquids.[^21^, ^46^] Miran et al. described PILs based on 1,8‐diazabicyclo[5,4,0]‐7‐ene (DBU) and different anions, with the triflate based ILs showing slightly higher conductivities than their mesylate counterparts.^[63]^ Gruzdev et al. showed that triethanolamine‐based ILs also have higher ionic conductivities with TfO anions than when mesylate anions are used.^[59]^

Moreover, we also observe an influence of the alkyl chain length on the conductivities: the ILs with the hexyl chains show higher conductivities than their counterparts with octyl chains. This behavior is more pronounced at high temperatures than at RT. This (slight) difference in the conductivities probably originates in the shorter alkyl chains and therefore higher ionic mobility of the hexylammonium ions in THMS and THOTf.

At elevated temperatures, the IGs show ionic conductivities that are comparable to those of the parent ILs. At room temperature, however, the conductivities between IG and parent IL differ by almost one order of magnitude and the conductivities of the IGs are always lower than the conductivities of the neat ILs. The least effect is observed in THOTf and the respective IG THOTf_PR70.

When analyzing the conductivity over a wider temperature range, T_g_ of the respective systems can be observed and subsequently compared to the glass transition temperature found by DSC. Figure S13c shows the conductivities for all four mesylate based systems over a temperature range of 150 °C to up to −92 °C. All compounds exhibit Vogel‐Fulcher‐Tammann (VFT) behavior. The T_g_s can be distinguished by the Arrhenius crossover around 5.1 K^−1^ and can be more properly discerned by the Stickel analysis^[64]^ (Figure S14). This analysis is a derivative analysis of temperature variations of dynamic quantities which transforms VFT behavior into a linear dependence.[^65^, ^66^] This subsequently makes the determination of T_g_s determined via BDS more precisely accessible.

The comparisons between T_g_s determined via DSC (2^nd^ cooling run) and BDS can be found in Table 6. The T_g_s determined by BDS show the same trends as the ones found via DSC. TOMS shows the lowest T_g_, whereas the IGs show the highest temperatures, indicating a faster and easier transition into the glassy state upon cooling compared to the pure ILs. Interestingly though, the T_g_s compared for both ILs are very similar and a small difference is only seen for TOMS_PR70. Nevertheless, all glass transitions appear in a range of 7.3 K and are therefore very close to each other.

**Table 6:** T_g_ comparisons for mesylate based compounds.

Compound	Glass transition temperatures	
	T_g_ [°C] (DSC)^[a]^	T_g_ [°C] (BDS)
**THMS**	−79.3	−77.6
**THMS_PR70**	−73.3	−72.0
**TOMS**	−81.0	−84.1
**TOMS_PR70**	−79.2	−71.9

[a] T_g_ taken from 2^nd^ DSC cooling run.

As already shown in Figure 6, all ILs and IGs show non‐Arrhenius behavior indicated by a curvature of the graphs, which is typical for a wide range of ILs.[^30^, ^46^, ^67^, ^68^] This corresponds to a non‐linear dependence of conductivity on inverse temperature. Therefore, this behavior is best described with the VFT relation (Equation (2)). Here, σ_0_ represents the maximum ionic conductivity for infinite temperature, B is an adjustable parameter (or pseudo activation energy) and T_0_ is the ideal glass transition temperature.[^30^, ^59^, ^67^, ^69^, ^70^, ^71^]
(2)

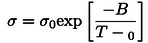




The values of the fitted parameters can be found in Table 7 and a graphical representation for the VFT fit of THMS and THMS_PR70 is shown in Figure S15. According to literature a dependence of the conductivity on the VFT equation hints at a liquid‐like nature of a system and a dependence of activation energies on segmental or site relaxation dynamics or the viscosity of a material rather than electrostatic or elastic contributions or transport via ion hopping.[^52^, ^72^, ^73^]

**Table 7:** VFT‐fitting parameters for the pure ILs and IGs.

VFT‐fitting parameters			
Compound	$\sigma_{0}$ [S/cm]	B [K]	T_0_ [K]
**THMS**	2.9×10^−1^	654.1	140.6
**THMS_PR70**	2.7×10^−1^	735.4	143.4
**TOMS**	2.1×10^−1^	697.9	131.9
**TOMS_PR70**	2.4×10^−1^	841.0	133.1
**THOTf**	2.7×10^−1^	584.5	144.0
**THOTf_PR70**	3.3×10^−1^	724.3	137.2
**TOOTf**	1.9×10^−1^	653.2	139.6
**TOOTf_PR70**	6.6×10^−2^	651.9	143.6

The pseudo activation energies B (which are related to segmental mobility^[74]^) for all four ILs follow the same trend as previously seen for the ionic conductivities. The triflate based ILs have slightly lower energy values compared to their mesylate counterparts and the octyl‐based ILs show slightly higher ones than the hexyl‐based ones. All apparent activation energies in this study are comparable with activation energies calculated by VFT analysis for other ILs and IL based electrolytes.[^25^, ^30^, ^46^, ^52^, ^53^, ^67^, ^68^, ^69^, ^70^]

**Figure 7:**
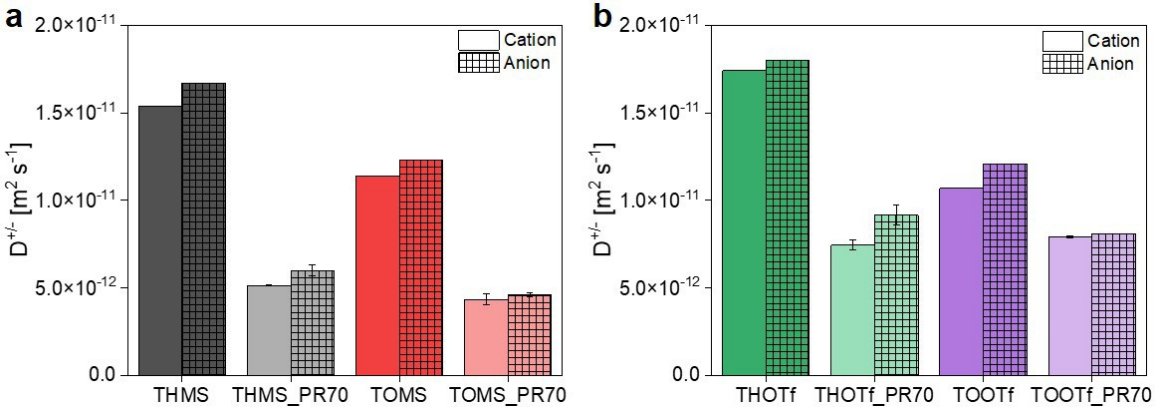
Cation (blank columns) and anion (patterned columns) diffusion coefficients for a) mesylate based and b) triflate based compounds.

For a more detailed insight into the behavior and transport properties of the ILs and IGs at elevated temperatures, diffusion coefficients of the ionic components in both systems were determined at 60 °C using ^1^H and ^19^F pulsed field gradient (PFG−) NMR spectroscopy, Figure 7. A temperature of 60 °C was chosen, as higher temperatures introduced measurements problems for the ILs due to the beginning of convection, while lower temperatures did not yield a sufficient echo decay for the slow diffusion in the IGs.

Clearly, in all compounds the anion diffusion coefficient D^−^ is higher than the cation diffusion coefficient D^+^. This can be expected given the different ion sizes of cation and anion. Both cations are significantly more bulky and therefore slower than the triflate or mesylate anions. Formation of cation aggregates, which will be discussed below, might also slow down the cation diffusion. D^−^ can, however, also be smaller than D^+^ when the anion is paired with the smaller triethyl‐ammonium cation, as several groups have shown for TEMS and TEOTf.[^21^, ^43^, ^46^, ^75^] Overall, the diffusion coefficients reflect the trends observed for the ionic conductivities of the pure ILs, namely: 1.) fluorinated compounds have higher D^+/−^ than mesylate based compounds, 2.) shorter chains, i. e. smaller molecules, lead to higher D^+/−^ than longer chains and larger molecules. For the most part both diffusion coefficients follow the trend THOTf>THMS>TOMS≈TOOTf for the pure ILs and are in the range of other mesylate or triflate based ILs.[^21^, ^43^, ^46^, ^47^, ^63^] The overall slightly lower diffusion coefficients for the ILs investigated in this study can, as already discussed, be assigned to the significantly longer alkyl‐chains, when comparing C_2_ (triethyl) to C_6_‐ or C_8_‐carbon chain‐based compounds.

Incorporation of the ILs into the polymer matrix causes a decrease in both diffusion coefficients D^+/−^, which is again in agreement with the conductivity data. The data seems to suggest, that cation diffusion in TOOTf_PR70 is the same or even slightly faster than in THOTf_PR70. It must however be considered in this case that D^+^ in TOOTf_PR70 may also be affected by some phase separation in these IGs.

Moreover, the differences between diffusion in the pure ILs and the IGs are smaller for the octyl‐based ILs. The incorporation of the ILs into the polymer matrix shows a stronger influence on the overall diffusion in THOTf and THMS than on their longer‐chained counterparts. This might be explained by the overall composition of the ILs themselves. In the octyl‐chained ILs the ions generally experience a lower self‐diffusion due to the long alkyl‐chains on the cations. They therefore have smaller diffusion coefficients even in the pure ILs. Besides the larger size of the cation this might also be due to the formation of micelles or micelle‐like aggregates, in which the chains separate from the ionic groups. Incorporation into the polymer could then cause (partial) disintegration of these aggregates due to interactions of the cation with the resin. Thus, the differences in diffusion coefficients between the hexyl (THMS, THOTf) and octyl (TOMS, TOOTf) compounds are reduced in the respective IGs. Consistent with this idea it has already been shown that in ILs containing cations with chain lengths of n=5 aggregation starts to play a role for IL properties such as diffusion or transference numbers.[^76^, ^77^] This observation is especially distinct for the cations. As the same trend cannot be observed in the ionic conductivities, this suggests a higher portion of anion conductivity.

To assess the relevance of charge transport based on the acidic proton, the spectral line of the latter must be analyzed separately and its diffusion coefficient compared to that of the non‐acidic protons on the cation. This can be performed in many other protic ILs, e. g. ILs based on triethylamine.^[75]^ In the present ILs, however, the acidic proton resonance is visible in standard 1H‐NMR measurements of the bulk ILs, but it disappears in diffusion measurements. Spin relaxation measurements show a very short T_2_ (ca. 230 μs) for the acidic proton, which is too short to allow detection in a diffusion measurement, where an observation time of at least several ms is required between the gradient pulses. T_1_ in contrast shows typical values (ca. 550 ms). This finding is interesting, because the large difference between T_1_ and T_2_ implies very slow dynamics of the acidic proton.

To estimate the ionicities of the different ILs based on the NMR results, calculation of the molar conductivity from the BDS data is necessary. The molar conductivity Λ_Imp_ of the ILs can be extracted by applying the following relation:

(3) $$\Lambda_{Imp}=\sigma \cdot \;\frac{{MW}_{IL}}{\rho }$$

Herein σ is the ionic conductivity determined via impedance spectroscopy, MW the molecular weight of the ionic liquid (ion pair) and ρ the sample density (Table S3). The Nernst‐Einstein‐equation supplies an estimated ionic conductivity Λ_NMR_ based on the PFG NMR experiments, assuming independent motion of cation and anion without any correlations.^[46]^

(4) $$\Lambda_{NMR}=\frac{N_{A}e^{2}}{k_{B}T}\cdot \left(D^{+}+D^{-}\right)$$

The ratio Λ_Imp_/Λ_NMR_ between these two molar conductivities is described as the ionicity, where values close to unity suggest full ion dissociation, whereas values smaller than unity describe systems where charge transport is impaired by ion correlations, for example formation of ion pairs or aggregates. Table 8 summarizes the necessary values to estimate the ionicities for the investigated compounds at 60 °C. The resulting ionicities range around 0.04 for the mesylate and 0.08 for the triflate based ILs (Table 9). These values are comparable to some ionicities found for other PILs with acetate or trifluoroacetate as anions.[^78^, ^79^]

**Table 8:** Diffusion and conductivity data at 60 °C.

Diffusion data at 60 °C				Conductivity data at 60 °C	
Compound	D^+^ [10^−11^ m^2^ s^−1^]	D^−^ [10^−11^ m^2^ s^−1^]	$\Lambda_{NMR}$ [S cm^2^/mol]	σ [10^−4^ S/cm]	$\Lambda_{Imp}$ [S cm^2^/mol]
**THMS**	1.54	1.67	1.079	1.20	0.05
**THMS_PR70**	0.51	0.60	0.375	0.38	‐
**TOMS**	1.14	1.23	0.796	0.75	0.04
**TOMS_PR70**	0.43	0.46	0.300	0.14	‐
**THOTf**	1.74	1.80	1.190	2.20	0.09
**THOTf_PR70**	0.74	0.92	0.558	0.68	‐
**TOOTf**	1.07	1.21	0.766	0.79	0.04
**TOOTf_PR70**	0.79	0.81	0.538	0.24	‐

**Table 9:** Ionicities of the PILs.

Compound	Ionicity [$\Lambda_{Imp}$ /$\Lambda_{NMR}$ ]
**THMS**	0.043
**TOMS**	0.047
**THOTf**	0.077
**TOOTf**	0.053

Overall, the ionicities calculated for the PILs in this study are quite low compared to the ionicities of other mesylate or triflate containing compounds.[^46^, ^78^] However, a higher ionicity for the triflate based ILs in comparison to the mesylate systems is in good agreement with literature when comparing mesylate to triflate PILs.[^46^, ^78^] However, the generally low ionicities again suggest a considerable contribution of ion pairs to the structures of the ILs, which has already been discussed above. Strong cation‐anion correlations have already been found for the shorter chained triethylammonium triflate (TEOTf), where the ionicity value was 0.5, even though proton transfer was almost complete. In contrast to the triethyl based ILs, the local separation of ionic groups from hydrophobic hexyl and octyl chains for the ILs investigated here might lead to an even stronger association of the ionic groups, see the discussion of hydrophobic aggregates above. Therefore, the ionic groups might associate even more strongly with each other than in triethyl based ILs, leading to rather stable aggregates, which are not only structurally relevant, but influence transport, as aggregates might show high diffusion coefficients, but low ionicities. Moreover, the anions in these long‐chained ILs have even less possibilities for single anion charge transport, as the pathways are blocked by relatively large hydrophobic regions, leading to transport occurring in form of pairs or larger aggregates. It must be noted however, that the concept of “ionicity” is currently being discussion in the literature for protic ILs.[^46^, ^80^, ^81^] It is therefore likely that a better understanding of the ionicity will emerge in the future.

**Figure 8:**
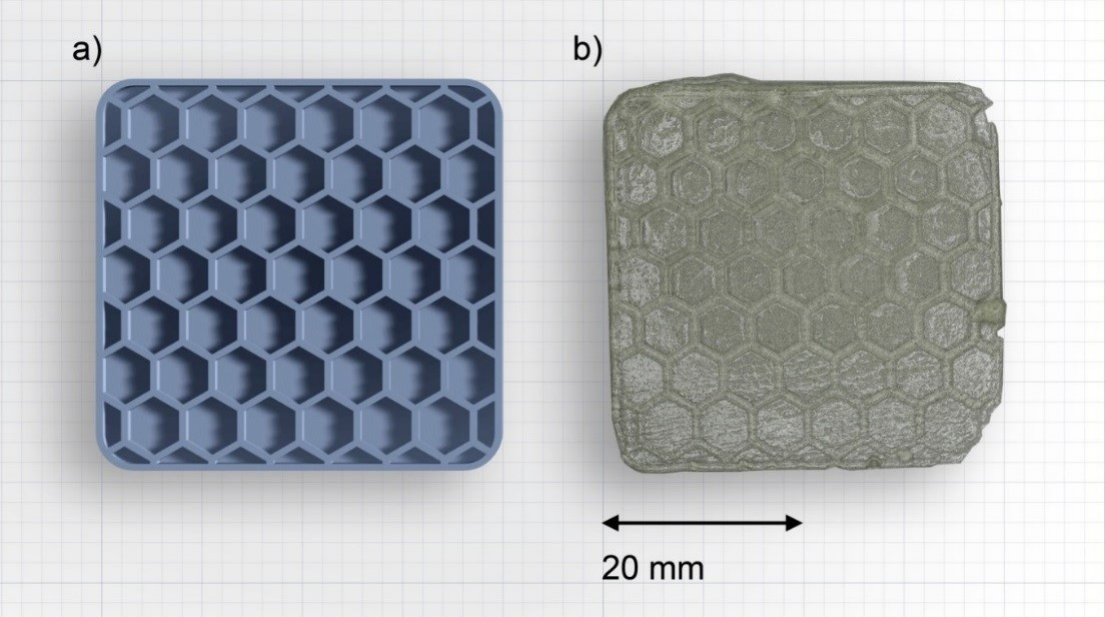
Starting file used for printing (a), image of the printed object THMS_PR70_SLA (b).

In our previous work it was possible to prepare a 3D honeycomb structure from a mixture of 60 wt % of a pure protic IL 1‐butyl‐3‐methyl‐imidazoliumbutylsulfonic acid tosylate [BmimSO_3_H][pTS] with a commercial clear photoreactive resin using an SLA printer.^[26]^ The final print was limited by the amount of IL that could be incorporated into the matrix. In contrast, the new IL THMS introduced here shows a much better miscibility with the SLA resin; this might be due to the more hydrophobic nature of the ILs investigated in this study in comparison to the zwitterionic ILs of the previous one. Indeed, Figure 8 shows that the final printed IG membrane using 70 % of THMS as the IL component is very uniform, translucent and is a quite faithful reproduction of the original print file. Considering that SLA printing of IGs is still in its infancy the results are very promising and will be further adapted to the material system in the future.

**Figure 9:**
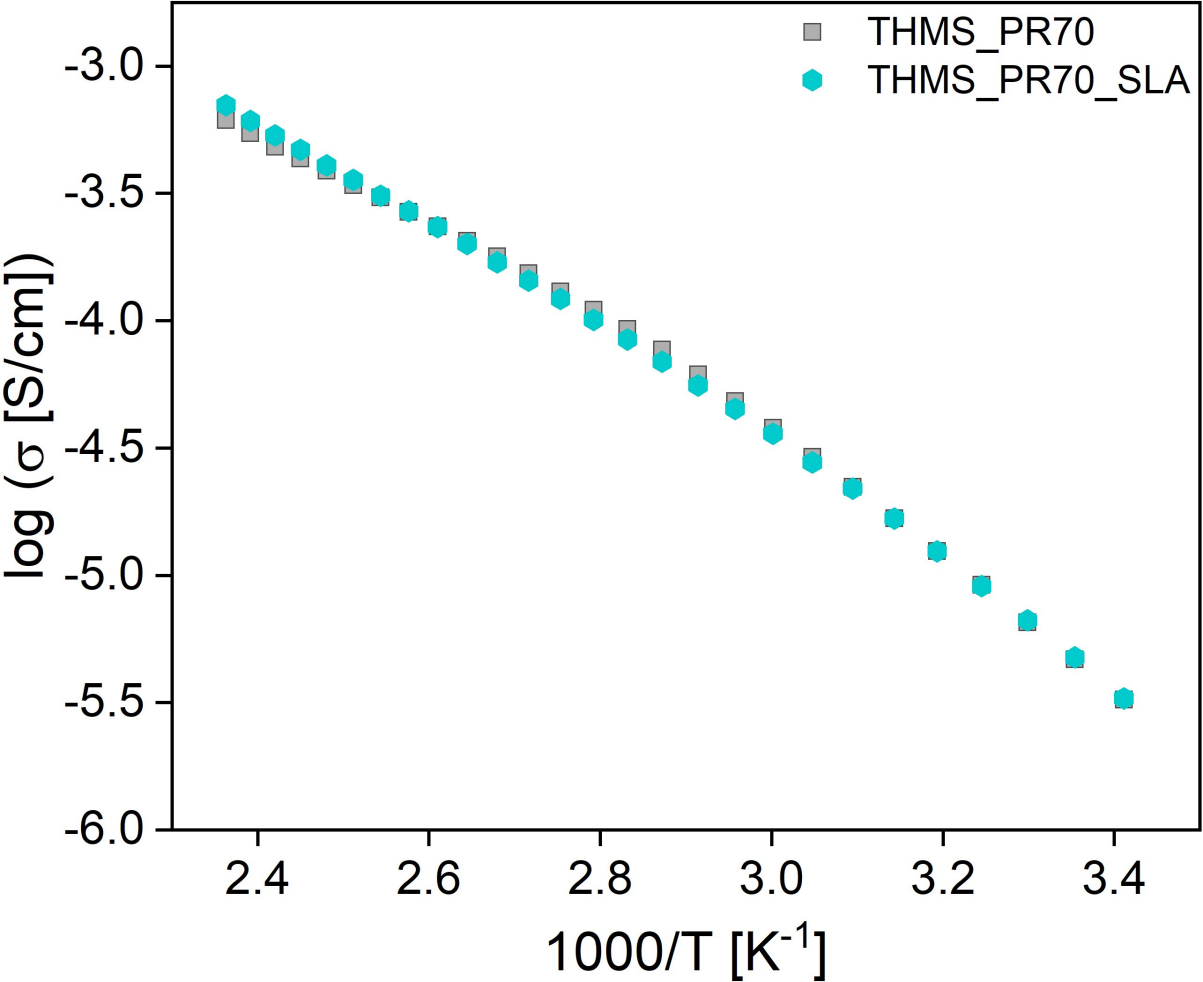
Arrhenius plot of data obtained from a 3D printed THMS_PR70_SLA with smooth surface and non‐printed (that is, a cast and cured) THMS_PR70 IG. Note that the conductivity results shown were obtained from an SLA‐printed IG with a flat surface (not with a honeycomb structure). The honeycomb IG could not be measured due to its structured surface, as the contact area of the IG is taken into account to calculate the conductivity.

To demonstrate that the SLA printing process produces properties that are comparable to those of the IGs described in the first part of the article, we have again measured the conductivities of the IG obtained from SLA printing and compared the data with the values obtained for the IG described before. Figure 9 shows the results of the conductivity measurements between 20 °C and 150 °C.

The ionic conductivity of the printed IG THMS_PR70_SLA is comparable to the values obtained from the cast and cured IG THMS_PR70 at the same temperatures. This suggests no significant differences between the structural nature of the cast and the printed IG and therefore a favorable organization of the IL inside the matrix material regardless of the specific preparation procedure.

Keeping in mind that the 3D printing process is not primarily designed for printing IGs the final product is very promising and acceptable for the development of a new product. Moreover, in light of the ongoing research in the field of 3D printing, the development of an even more conductive, thinner and 3D printable ionogel is only a matter of time. Overall, this work provides a promising prototype for these kinds of electrolytes and shows that micro structuring of these compounds is possible by means of (SLA) printing IGs. The importance of microstructures on the surface of fuel cell membranes is being highlighted by many groups, as they e. g. investigate the influence of patterned surfaces.[^82^, ^83^] It has been shown that wettability and overall surface characteristics can be improved by well‐defined patterns.^[84]^ 3D structures which increase the PEM/catalyst layer interface can also enhance mass transport and current density.^[85]^ Current work in our group and elsewhere[^28^, ^30^] is indeed concentrating on the development of 3D printable ionic liquids that can be polymerized to produce membranes without adding polymer resin. Preliminary experiments in the laboratory have already demonstrated that the process is scalable; the largest current experiment is based on about 0.5 L of the printing mixture. We currently do not see a reason why larger volumes should not be printable.

## Conclusions

3D printable ionogels are materials with a wide application potential. 3D printing hereby allows for an easy and application oriented preparation of, e. g., electrolyte materials with tailored properties, geometries and proportions. This study expands the current number of successfully printed IGs and presents IGs with up to 70 wt % of IL. Moreover, the ILs themselves, which are based on long‐chain tri‐n‐alklyamines and two different sulfonic acids (methanesulfonic and triflic acid), exhibit promising ionic conductivities at elevated temperatures and electrochemical stability windows of up to 3.4 V. This study also investigates the ion diffusion of ILs and IGs with D^+/−^ in the range of 10^−12^–10^−11^ m^2^ s^−1^. Ionicities of the presented ILs show comparatively low values despite these diffusion coefficients, leading to the assumption of a high portion of ion aggregates and possibly a fairly strong shielding of the ionic groups by the unpolar parts (alkyl chains) in the ILs. A further point that will require additional experiments is the qualitative observations that the ionogels remain stable and keep their properties over several years of storage in the laboratory.

## Conflict of Interests

The authors declare no conflict of interest.

1

## Supporting information

As a service to our authors and readers, this journal provides supporting information supplied by the authors. Such materials are peer reviewed and may be re‐organized for online delivery, but are not copy‐edited or typeset. Technical support issues arising from supporting information (other than missing files) should be addressed to the authors.

Supporting Information

## Data Availability

The data that support the findings of this study are available in the supplementary material of this article.

## References

[1] S. G. Chalk , J. F. Miller , J. Power Sources 2006, 159(1), 73–80. DOI: 10.1016/j.jpowsour.2006.04.058.

[2] M. Li , C. Wang , Z. Chen , K. Xu , J. Lu , Chem. Rev. 2020, 120(14), 6783–6819. DOI: 10.1021/acs.chemrev.9b00531. 32022546

[3] K. S. Ngai , S. Ramesh , K. Ramesh , J. C. Juan , Ionics 2016, 22(8), 1259–1279. DOI: 10.1007/s11581-016-1756-4.

[4] X. Cheng , J. Pan , Y. Zhao , M. Liao , H. Peng , Adv. Energy Mater. 2018, 8(7), 1702184. DOI: 10.1002/aenm.201702184.

[5] M.-A. Néouze , J. Le Bideau , P. Gaveau , S. Bellayer , A. I. Vioux , Chem. Mater. 2006, 18(17), 3931–3936. DOI: 10.1021/cm060656c. 17925968

[6] J. Le Bideau , L. Viau , A. I. Vioux , Chem. Soc. Rev. 2011, 40(2), 907–925. DOI: 10.1039/C0CS00059K. 21180731

[7] T. Torimoto , T. Tsuda , K. Okazaki , S. Kuwabata , Adv. Mater. 2010, 22(11), 1196–1221. DOI: 10.1002/adma.200902184. 20437507

[8] S. Werner , M. Haumann , P. Wasserscheid , Annu. Rev. Chem. Biomol. Eng. 2010, 1, 203–230. DOI: 10.1146/annurev-chembioeng-073009-100915. 22432579

[9] T. L. Greaves , C. J. Drummond , Chem. Rev. 2008, 108(1), 206–237. DOI: 10.1021/cr068040u. 18095716

[10] L. E. Shmukler , M. S. Gruzdev , N. O. Kudryakova , Y. A. Fadeeva , A. M. Kolker , L. P. Safonova , J. Mol. Liq. 2018, 266, 139–146. DOI: 10.1016/j.molliq.2018.06.059.

[11] V. Di Noto , E. Negro , J.-Y. Sanchez , C. Iojoiu , JACS 2010, 132(7), 2183–2195. DOI: 10.1021/ja906975z. 20102239

[12] S.-Y. Lee , A. Ogawa , M. Kanno , H. Nakamoto , T. Yasuda , M. Watanabe , JACS 2010, 132(28), 9764–9773. DOI: 10.1021/ja102367x. 20578771

[13] L. Zanchet , L. G. da Trindade , W. Bariviera , K. M. Nobre Borba , R. D. M. Santos , V. A. Paganin , C. P. de Oliveira , E. A. Ticianelli , E. M. A. Martini , M. O. de Souza , J. Mater. Sci. 2020, 55(16), 6928–6941. DOI: 10.1007/s10853-020-04454-4.

[14] T. Stettner , F. C. Walter , A. Balducci , Batteries Supercaps 2019, 2(1), 55–59. DOI: 10.1002/batt.201800096.

[15] S. Banerjee , D. E. Curtin , J. Fluor. Chem. 2004, 125(8), 1211–1216. DOI: 10.1016/j.jfluchem.2004.05.018.

[16] K. A. Mauritz , R. B. Moore , Chem. Rev. 2004, 104(10), 4535–4585. DOI: 10.1021/cr0207123. 15669162

[17] R. Souzy , B. Ameduri , Prog. Polym. Sci. 2005, 30(6), 644–687. DOI: 10.1016/j.progpolymsci.2005.03.004.

[18] O. Danyliv , A. Martinelli , J. Phys. Chem. C 2019, 123(23), 14813–14824. DOI: 10.1021/acs.jpcc.9b02874.

[19] J. Lu , F. Yan , J. Texter , Prog. Polym. Sci. 2009, 34(5), 431–448. DOI: 10.1016/j.progpolymsci.2008.12.001.

[20] W. Lu , K. Henry , C. Turchi , J. Pellegrino , J. Electrochem. Soc. 2008, 155(5), A361. DOI: 10.1149/1.2869202.

[21] M. Martinez , Y. Molmeret , L. Cointeaux , C. Iojoiu , J.-C. Leprêtre , N. El Kissi , P. Judeinstein , J.-Y. Sanchez , J. Power Sources 2010, 195(18), 5829–5839. DOI: 10.1016/j.jpowsour.2010.01.036.

[22] M. G. Cowan , M. Masuda , W. M. McDanel , Y. Kohno , D. L. Gin , R. D. Noble , J. Membr. Sci. 2016, 498, 408–413. DOI: 10.1016/j.memsci.2015.10.019.

[23] A. S. Shaplov , R. Marcilla , D. Mecerreyes , Electrochim. Acta 2015, 175, 18–34. DOI: 10.1016/j.electacta.2015.03.038.

[24] K. Matsumoto , T. Endo , Macromolecules 2008, 41(19), 6981–6986. DOI: 10.1021/ma801293j.

[25] M. A. B. H. Susan , T. Kaneko , A. Noda , M. Watanabe , JACS 2005, 127(13), 4976–4983. DOI: 10.1021/ja045155b. 15796564

[26] K. Zehbe , A. Lange , A. Taubert , Energy Fuels 2019, 33(12), 12885–12893. DOI: 10.1021/acs.energyfuels.9b03379.

[27] S. Pal , Y.-Z. Su , Y.-W. Chen , C.-H. Yu , C.-W. Kung , S.-S. Yu , ACS Appl. Mater. Interfaces. 2022, DOI: 10.1021/acsami.2c02690. 35604841

[28] Z. Wang , J. Zhang , J. Liu , S. Hao , H. Song , J. Zhang , ACS Appl. Mater. Interfaces 2021, 13(4), 5614–5624. DOI: 10.1021/acsami.0c21121. 33492940

[29] M. Zhang , X. Tao , R. Yu , Y. He , X. Li , X. Chen , W. S. Huang , J. Mater. Chem. A 2022, 10(22), 12005–12015. DOI: 10.1039/d1ta09641a.

[30] S. Sen , S. E. Goodwin , P. V. Barbará , G. A. Rance , D. Wales , J. M. Cameron , V. Sans , M. Mamlouk , K. Scott , D. A. Walsh , ACS Appl. Polym. Mater. 2021, 3(1), 200–208. DOI: 10.1021/acsapm.0c01042.

[31] L. Zhao , F. Ran , Chem. Commun. 2023, 59(46), 6969–6986. DOI: 10.1039/d3cc00412k. 37165689

[32] X. Yu , A. Manthiram , Energy Environ. Sci. 2018, 11(3), 527–543. DOI: 10.1039/C7EE02555F.

[33] X. Wang , J. Chen , D. Wang , Z. Mao , Nat. Commun. 2021, 12(1), 7109. DOI: 10.1038/s41467-021-27473-4. 34876588 PMC8651668

[34] S. Zhang , S. Xue , Y. Wang , G. Zhang , N. Arif , P. Li , Y.-J. Zeng , Batteries 2023, 9(11), 546, DOI: 10.3390/batteries9110546.

[35] S. Ponnada , D. Babu Gorle , R. S. Chandra Bose , M. Sadat Kiai , M. Devi , C. Venkateswara Raju , N. Baydogan , K. Kar Nanda , F. Marken , R. K. Sharma , Batteries Supercaps 2022, 5(8). DOI: 10.1002/batt.202200223.

[36] M. P. Browne , E. Redondo , M. Pumera , Chem. Rev. 2020, 120(5), 2783–2810. DOI: 10.1021/acs.chemrev.9b00783. 32049499

[37] M. Cheng , R. Deivanayagam , R. Shahbazian-Yassar , Batteries Supercaps 2020, 3(2), 130–146. DOI: 10.1002/batt.201900130.

[38] F. Zhang , M. Wei , V. V. Viswanathan , B. Swart , Y. Shao , G. Wu , C. Zhou , Nano Energy 2017, 40, 418–431. DOI: 10.1016/j.nanoen.2017.08.037.

[39] Q. Chen , R. Xu , Z. He , K. Zhao , L. Pan , J. Electrochem. Soc. 2017, 164(9), A1852–A1857. DOI: 10.1149/2.0651709jes.

[40] V. Di Noto , M. Piga , G. A. Giffin , S. Lavina , E. S. Smotkin , J.-Y. Sanchez , C. Iojoiu , J. Phys. Chem. C 2012, 116(1), 1361–1369. DOI: 10.1021/jp204241y.

[41] V. Di Noto , M. Piga , G. A. Giffin , S. Lavina , E. S. Smotkin , J.-Y. Sanchez , C. Iojoiu , J. Phys. Chem. C 2012, 116(1), 1370–1379. DOI: 10.1021/jp204242q.

[42] Z. Wojnarowska , A. Lange , A. Taubert , M. Paluch , ACS Appl. Mater. Interfaces 2021, 13(26), 30614–30624. DOI: 10.1021/acsami.1c06260. 34164974 PMC8289238

[43] C. Iojoiu , M. Martinez , M. Hanna , Y. Molmeret , L. Cointeaux , J.-C. Leprêtre , N. E. Kissi , J. Guindet , P. Judeinstein , J.-Y. Sanchez , Polym. Adv. Technol. 2008, 19(10), 1406–1414. DOI: 10.1002/pat.1219.

[44] H. Nakamoto , M. Watanabe , Chem. Commun. 2007(24), 2539–2541. DOI: 10.1039/B618953A. 17563822

[45] E. O. Stejskal , J. E. Tanner , J. Chem. Phys. 1965, 42(1), 288–292. DOI: 10.1063/1.1695690.

[46] A. Mariani , M. Bonomo , X. Gao , B. Centrella , A. Nucara , R. Buscaino , A. Barge , N. Barbero , L. Gontrani , S. Passerini , J. Mol. Liq. 2021, 324, 115069. DOI: 10.1016/j.molliq.2020.115069.

[47] J. L. Lebga-Nebane , S. E. Rock , J. Franclemont , D. Roy , S. Krishnan , Ind. Eng. Chem. Res. 2012, 51(43), 14084–14098. DOI: 10.1021/ie301687c.

[48] T. Wu , L.-S. Jou , Y.-C. Lin , S.-G. Su , I.-W. Sun , ECS Trans. 2011, 33(26), 83–86. DOI: 10.1149/1.3557878.

[49] U. A. Rana , R. Vijayaraghavan , M. Walther , J. Sun , A. A. J. Torriero , M. Forsyth , D. R. MacFarlane , Chem. Commun. 2011, 47(42), 11612–11614. DOI: 10.1039/C1CC14761G. 21963830

[50] K. Elamin , M. Shojaatalhosseini , O. Danyliv , A. Martinelli , J. Swenson , Electrochim. Acta 2019, 299, 979–986. DOI: 10.1016/j.electacta.2018.12.154.

[51] S. Zhang , K. H. Lee , J. Sun , C. D. Frisbie , T. P. Lodge , Macromolecules 2011, 44(22), 8981–8989. DOI: 10.1021/ma201356j.

[52] H. Pan , J. Luo , J. Zhao , M. Wubbenhorst , IEEE Trans. Dielect. Electr. Insul. 2022, 1. DOI: 10.1109/TDEI.2022.3164064.

[53] J. Luo , O. Conrad , I. F. J. Vankelecom , J. Mater. Chem. 2012, 22(38), 20574. DOI: 10.1039/C2JM34359B.

[54] A. Gallastegui , F. Foglia , P. F. McMillan , N. Casado , A. Gueguen , D. Mecerreyes , Polymer 2023, 280, 126064. DOI: 10.1016/j.polymer.2023.126064.

[55] G. P. Pandey , S. A. Hashmi , J. Power Sources 2009, 187(2), 627–634. DOI: 10.1016/j.jpowsour.2008.

[56] N. De Vos , C. Maton , C. V. Stevens , ChemElectroChem 2014, 1(8), 1258–1270. DOI: 10.1002/celc.201402086.

[57] M. Hayyan , F. S. Mjalli , M. A. Hashim , I. M. AlNashef , T. X. Mei , J. Ind. Eng. Chem. 2013, 19(1), 106–112. DOI: 10.1016/j.jiec.2012.07.011.

[58] S. Kazemiabnavi , Z. Zhang , K. Thornton , S. Banerjee , J. Phys. Chem. B 2016, 120(25), 5691–5702. DOI: 10.1021/acs.jpcb.6b03433. 27266487

[59] M. S. Gruzdev , L. E. Shmukler , N. O. Kudryakova , A. M. Kolker , Y. Sergeeva , L. P. Safonova , J. Mol. Liq. 2017, 242, 838–844. DOI: 10.1016/j.molliq.2017.07.078.

[60] A. Khan , X. Lu , L. Aldous , C. Zhao , J. Phys. Chem. C 2013, 117(36), 18334–18342. DOI: 10.1021/jp405759j.

[61] K. Kakinuma , H. Taniguchi , T. Asakawa , T. Miyao , M. Uchida , Y. Aoki , T. Akiyama , A. Masuda , N. Sato , A. Iiyama , J. Electrochem. Soc. 2022, 169(4), 44522. DOI: 10.1149/1945-7111/ac624b.

[62] T. Xiao , R. Wang , Z. Chang , Z. Fang , Z. Zhu , C. Xu , Prog. Nat. Sci.: Mater. Int. 2020, 30(6), 743–750. DOI: 10.1016/j.pnsc.2020.08.014.

[63] M. S. Miran , H. Kinoshita , T. Yasuda , M. A. B. H. Susan , M. Watanabe , Phys. Chem. Chem. Phys. 2012, 14(15), 5178–5186. DOI: 10.1039/c2cp00007e. 22415497

[64] F. Stickel , E. W. Fischer , R. Richert , J. Chem. Phys. 1995, 102(15), 6251–6257. DOI: 10.1063/1.469071.

[65] M. Musiał , S. Cheng , Z. Wojnarowska , M. D. Paluch , J. Mol. Liq. 2020, 317, 113971. DOI: 10.1016/j.molliq.2020.113971.

[66] B. Yao , M. Paluch , Z. Wojnarowska , Sci. Rep. 2023, 13(1), 3040. DOI: 10.1038/s41598-023-29518-8. 36810358 PMC9944924

[67] I. Bandrés , D. F. Montaño , I. Gascón , P. Cea , C. Lafuente , Electrochim. Acta 2010, 55(7), 2252–2257. DOI: 10.1016/j.electacta.2009.11.073.

[68] J. Vila , P. Ginés , J. M. Pico , C. Franjo , E. Jiménez , L. M. Varela , O. Cabeza , Fluid Phase Equilib. 2006, 242(2), 141–146. DOI: 10.1016/j.fluid.2006.01.022.

[69] I. Abdurrokhman , K. Elamin , O. Danyliv , M. Hasani , J. Swenson , A. Martinelli , J. Phys. Chem. B 2019, 123(18), 4044–4054. DOI: 10.1021/acs.jpcb.9b01274. 30995045

[70] S. Noor , P. M. Bayley , M. Forsyth , D. R. MacFarlane , Electrochim. Acta 2013, 91, 219–226. DOI: 10.1016/j.electacta.2012.11.113.

[71] J. Vila , C. Franjo , J. M. Pico , L. M. Varela , O. Cabeza , Port. Electrochim. Acta 2007, 25(1), 163–172. DOI: 10.4152/pea.200701163.

[72] X. Tang , X. Chang , B. Zhu , L. Cui , B. Jiang , F. Meng , G. Yan , Polym. Adv. Technol. 2022, 33(12), 4317–4329. DOI: 10.1002/pat.5861.

[73] Q. Ruan , M. Yao , Y. Du , H. Dong , J. Liu , X. Yuan , W. Fang , G. Zhao , H. Zhang , Nano Energy 2023, 106, 108087. DOI: 10.1016/j.nanoen.2022.108087.

[74] S. Wang , X. Liu , A. Wang , Z. Wang , J. Chen , Q. Zeng , X. Wang , L. Zhang , Polym. Chem. 2018, 9(37), 4674–4682. DOI: 10.1039/c8py00951a.

[75] M. Middendorf , M. Schönhoff , J. Phys. Chem. B 2024, 128(12), 2939–2947. DOI: 10.1021/acs.jpcb.3c08156. 38484313

[76] M. Kunze , S. Jeong , E. Paillard , M. Schönhoff , M. Winter , S. Passerini , Adv. Energy Mater. 2011, 1(2), 274–281. DOI: 10.1002/aenm.201000052.

[77] M. Kunze , E. Paillard , S. Jeong , G. B. Appetecchi , M. Schönhoff , M. Winter , S. Passerini , J. Phys. Chem. C 2011, 115(39), 19431–19436. DOI: 10.1021/jp2055969.

[78] S. K. Davidowski , F. Thompson , W. Huang , M. Hasani , S. A. Amin , C. A. Angell , J. L. Yarger , J. Phys. Chem. B 2016, 120(18), 4279–4285. DOI: 10.1021/acs.jpcb.6b01203. 27088704

[79] S. K. Mann , S. P. Brown , D. R. MacFarlane , ChemPhysChem 2020, 21(13), 1444–1454. DOI: 10.1002/cphc.202000242. 32445198

[80] K. R. Harris , J. Phys. Chem. B 2019, 123(32), 7014–7023. DOI: 10.1021/acs.jpcb.9b04443. 31318219

[81] D. R. MacFarlane , M. Forsyth , E. I. Izgorodina , A. P. Abbott , G. Annat , K. Fraser , Phys. Chem. Chem. Phys. 2009, 11(25), 4962–4967. DOI: 10.1039/b900201d. 19562126

[82] S. Jang , Y. S. Kang , D. Kim , S. Park , C. Seol , S. Lee , S. M. Kim , S. J. Yoo , Adv. Mater. 2023, 35(43), e2204902. DOI: 10.1002/adma.202204902. 36222387

[83] Y. Ke , W. Yuan , F. Zhou , W. Guo , J. Li , Z. Zhuang , X. Su , B. Lu , Y. Zhao , Y. Tang , Y. Chen , J. Song , Renew. Sustain. Energy Rev. 2021, 145, 110860. DOI: 10.1016/j.rser.2021.110860.

[84] R. Umezaki , J. Murata , Mater. Chem. Phys. 2021, 259, 124081. DOI: 10.1016/j.matchemphys.2020.124081.

[85] M. Chen , C. Zhao , F. Sun , J. Fan , H. Li , H. Wang , eTransportation 2020, 5, 100075. DOI: 10.1016/j.etran.2020.100075.

