# Supplementary material for: 3D Printable Polymer Electrolytes for Ionic Conduction based on Protic Ionic Liquids
Source: Chemphyschem. 2025 Jan 8;26(4):e202400849. doi: 10.1002/cphc.202400849 (PMC11832060; doi:10.1002/cphc.202400849)
Supplement: Supplementary file 1 — Supporting Information [file CPHC-26-e202400849-s001.pdf]

ChemPhysChem

Supporting Information

3D Printable Polymer Electrolytes for Ionic Conduction based on Protic Ionic Liquids

Alyna Lange,* Sajal Arwish, Aurelie Rensonnet, Khalid Elamin, Iqbaal Abdurrokhman, Zaneta Wojnarowska, Mark Rosenwinkel, Cedric Malherbe, Monika Schönhoff, Kerstin Zehbe, and Andreas Taubert*

3D Printable Polymer Electrolytes for Ionic Conduction based on Protic Ionic Liquids

Alyna Lange[1]*, Sajal Arwish[2], Aurelie Rensonnet[3], Khalid Elamin[4], Iqbaal Abdurrokhman[4], Zaneta Wojnarowska[5], Mark Rosenwinkel[2], Cedric Malherbe[3], Monika Schönhoff[2], Kerstin Zehbe[1], Andreas Taubert[1]*

1. Batch reproducibility

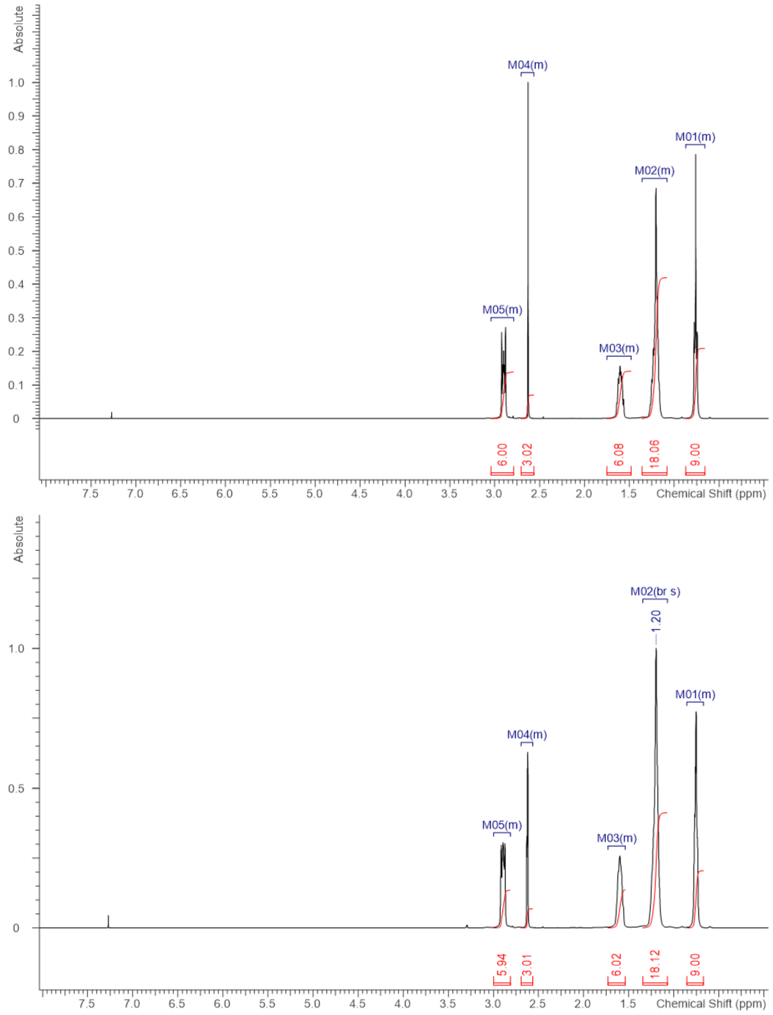

Figure S1: ^{1}H-NMR spectra for two different THMS batches.

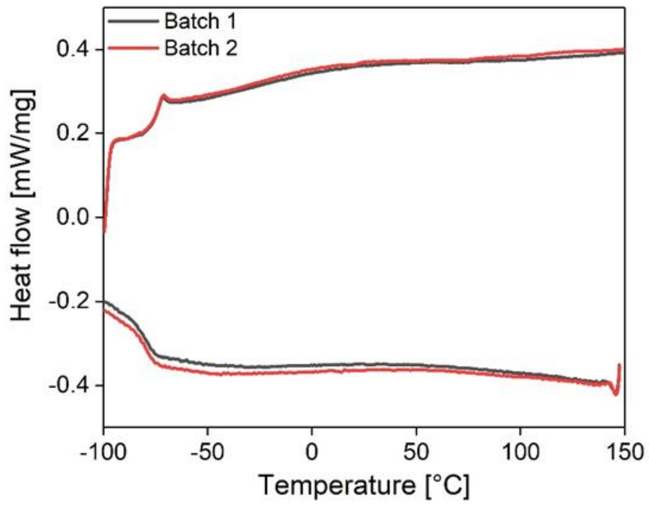

Figure S2: 2nd heating and cooling DSC runs for two different THMS batches.

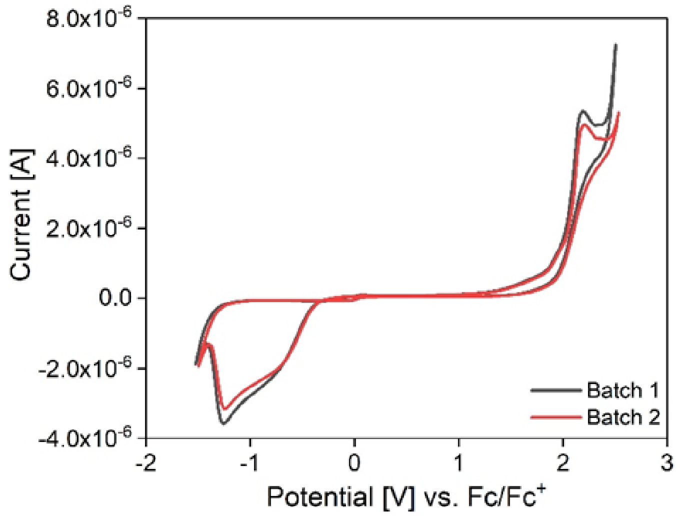

Figure S3: CV curves for two different THMS batches.

2. Ionogel images

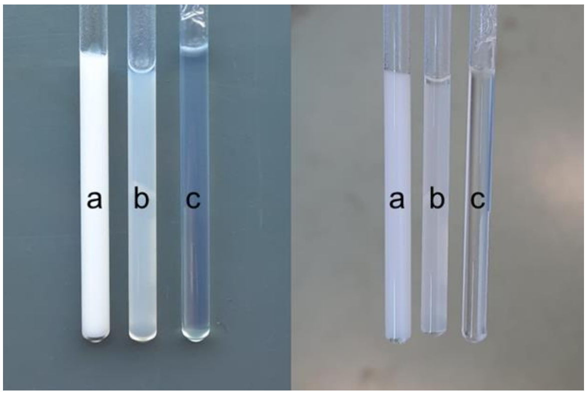

Figure S4: Representative pictures of selected IGs with different compatibilities: a) poor compatibility (TOOTf), b) limited compatibility (THOTf) and c) good compatibility (THMS).

3. Thermal data for ILs and IGs

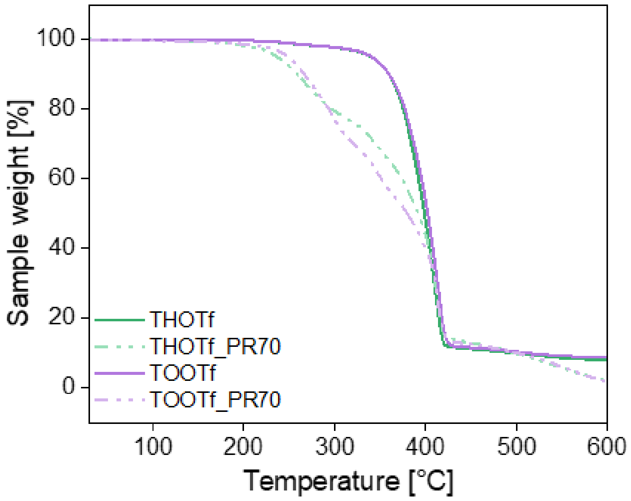

Figure S5: TGA curves for fluorinated ILs and IGs.

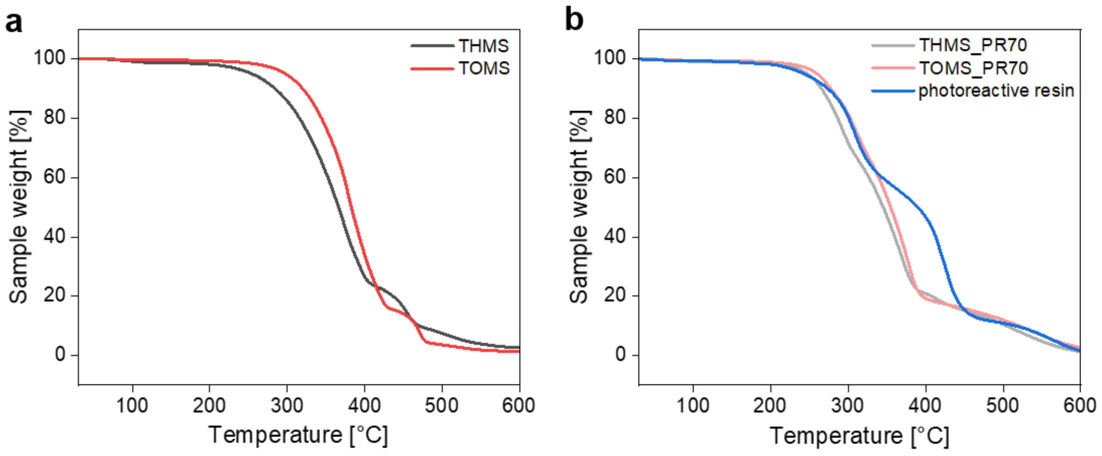

Figure S6: TGA curves for a) mesylate based ILs, b) mesylate based IGs and resin.

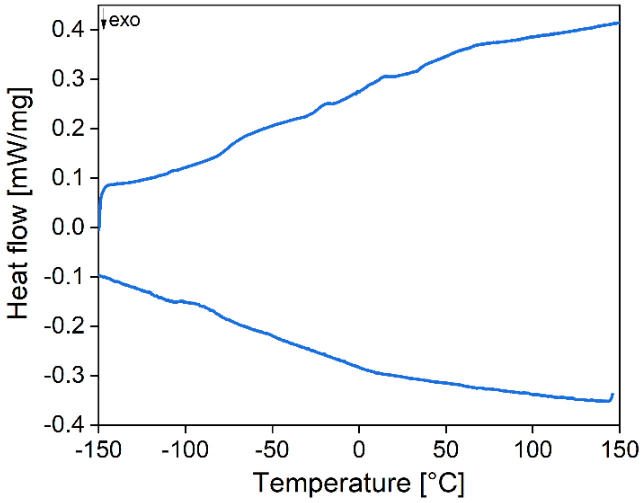

Figure S7: 2nd heating and cooling DSC runs for photoreactive resin.

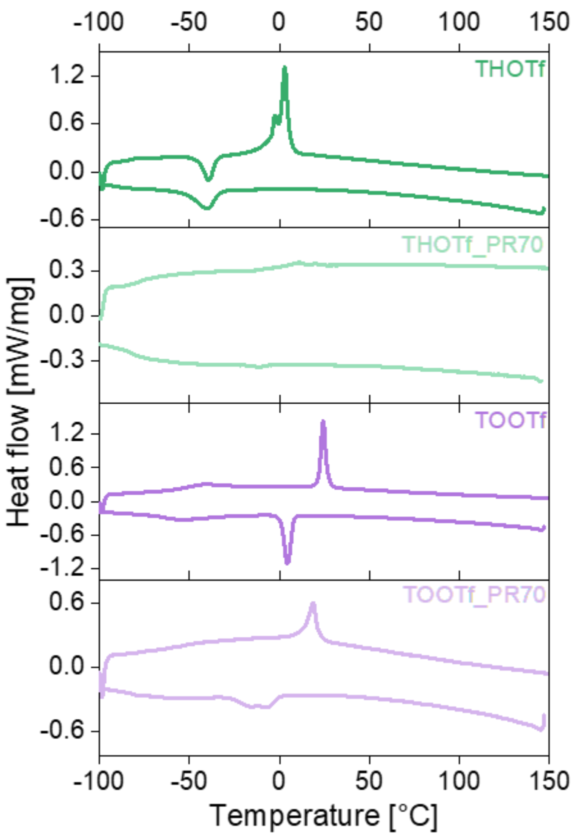

Figure S8: 2nd heating and cooling DSC runs for fluorinated ILs and IGs.

4. Detailed IR discussion

ILs: Bands in the range between 1470-1300 cm^{-1} can originate from different vibrations such as C-C- and N-C_3-asymmetric stretching or R-CH_2-R- and CH_3-bending. The pronounced bands between 1240-1140 arise from SO_3-asymmetric stretching and therefore show the presence of sulfonate groups. These can also be identified via the SO_3-symmetric stretching bands at 1040-1030 cm^{-1} and the SO_3-bending vibrations between 550-520 cm^{-1}. The presence of both methanesulfonate and trialkylammonium in the ILs is also confirmed by the presence of bands between 760-720 cm^{-1}. These can be assigned to C-S-stretching and N-C_3-symmetric stretching vibrations. [1]

IGs: The broad band at 3430 cm^{-1} is probably an overlapping signal from N-H- and O-H-vibrations, where the N-H-stretching vibrations originate from the polymer and the O-H-stretching vibrations from possible water uptake of the IG or interactions between polymer and IL. The band at 1730 cm^{-1} shows the presence of carbonyl groups via C=O-vibration. The band at 1540 cm^{-1} can be attributed to C-N-H bending and the band around 1370 cm^{-1} to C-N-stretching and C-H-bending/rocking vibrations. The presence of asymmetric C-O-C- and C-N-C-stretching vibrations originating in the acrylate- and urethane nature of the polymer-mixture can be seen in the pronounced band around 1110 cm^{-1}. Small bands around 1000 cm^{-1} can be attributed to symmetric C-O-C-stretching vibrations. [1–4] The characteristic bands between 1480-1440 cm^{-1} and 1230-1190 cm^{-1}, which can be associated with CH_2 and O-CH_3 bending and C-O stretching vibrations in the pure polymer resin cannot be observed clearly in the IGs, because they overlap with other bands from the ILs.

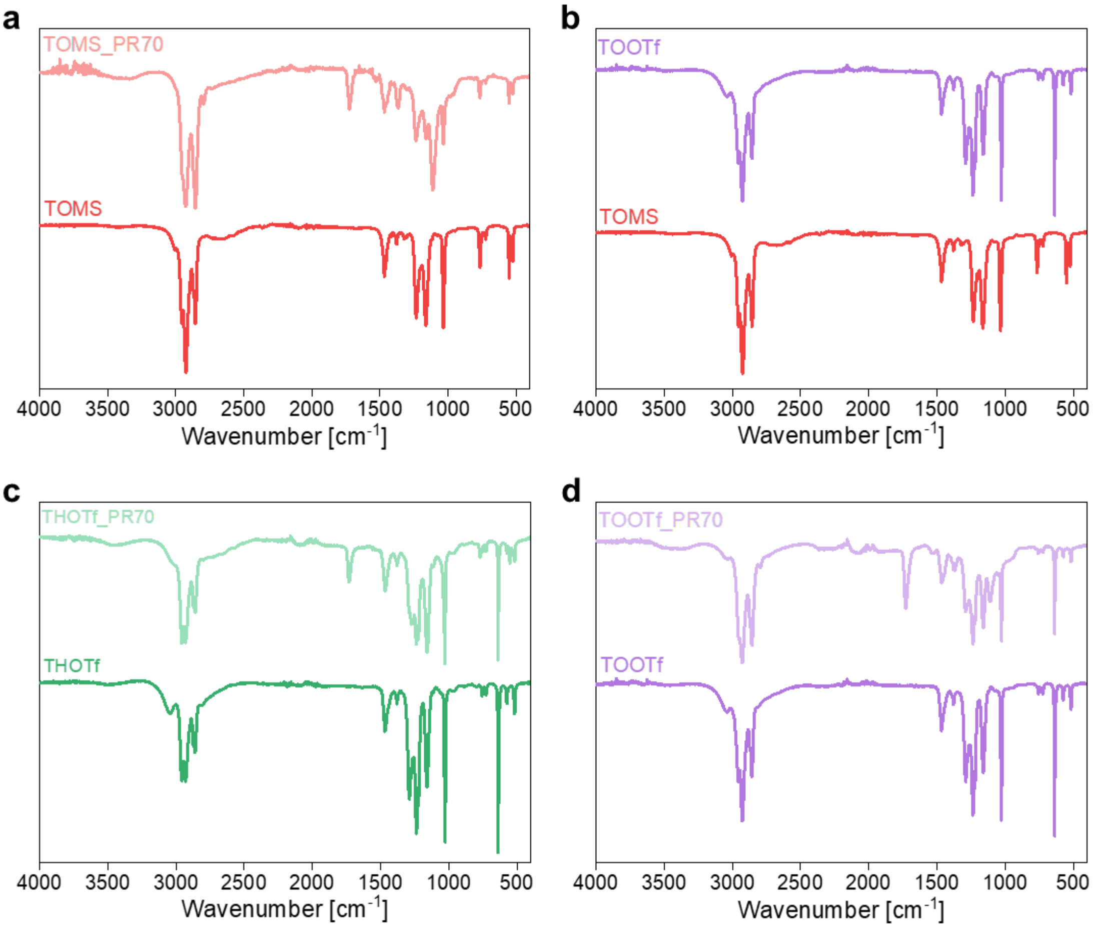

Figure S9: IR spectra; a) TOMS and IG, b) comparison trioctyl-based ILs, c) THOTf and IG, d) TOOTf and IG.

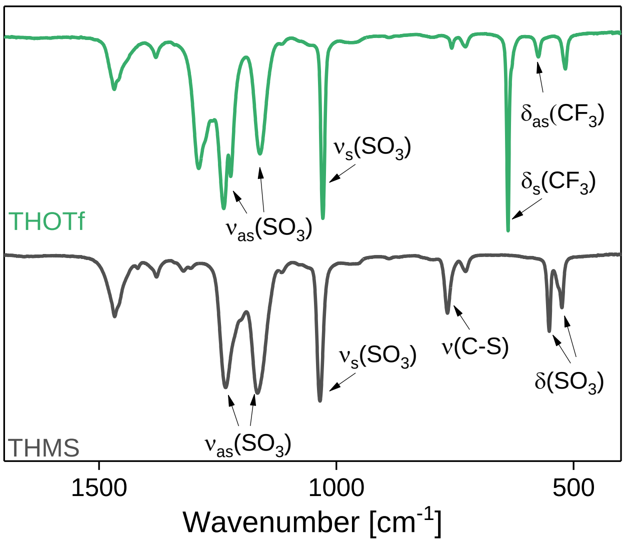

Figure S10: Fingerprint area of IR spectra for THMS and THOTf.

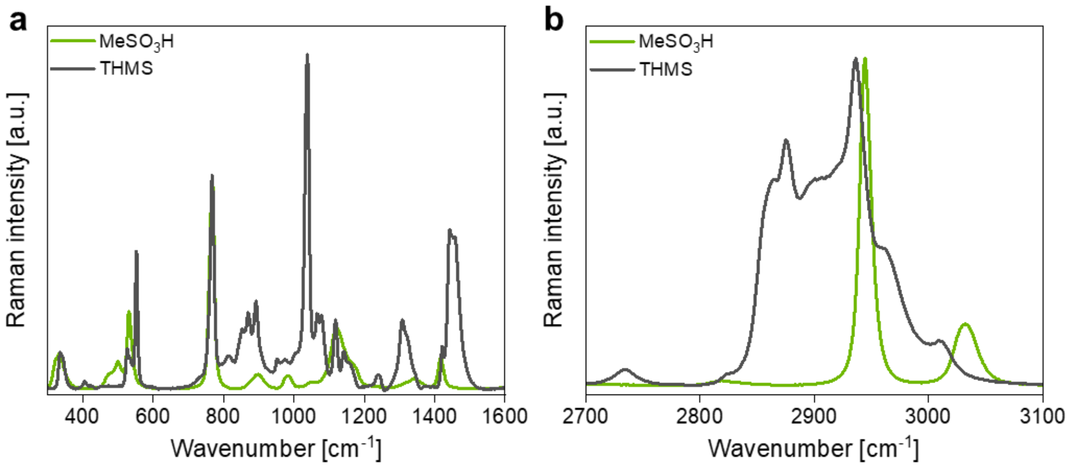

Figure S11: Raman spectra of THMS and pure methanesulfonic acid in a) low wavenumber region, and b) high wavenumber region.

Table S1: Raman shifts and assignments for THMS and TOMS.

**Raman shift [cm^{-1}]**		**Assignment**
**THMS**	**TOMS**	
340	340	SO_3 rocking[5,6]
530, 553	530, 553	SO_3 bending[5,6,9]
768	768	C-S stretching[5,6,9]
813, 851, 871, 892, 953	810, 846, 872, 891, 926, 958	C-C, C-N stretching, CH_2/CH_3 rocking[7,8,9]
1038	1039	SO_3 stretching[5,6,9]
1066, 1080	1063, 1081	
1118	1122	SO_3 stretching[9]
1141, 1159	1139, 1153	C-N stretching[9]
1307, 1315	1304, 1319	CH_2/CH_3 scissoring[7,8]
1421	1420	CH_3 bending (anion)[5,6,9]
1441, 1451	1439, 1453	CH_3/CH_2 bending[7,8,9]
2859	2855	Symmetric CH_2 stretching[9,10]
2876, 2895	2875, 2894	Symmetric CH_3 stretching[9,10]
2917	2913	Asymmetric CH_2 stretching[9,10]
2937, 2962	2937, 2962	Asymmetric CH_3 stretching[9,10]
3009	3009	Asymmetric N-H stretching[9]

Table S2: Raman shifts and assignments for pure $MeSO_3H$ acid.

Raman shift [cm^{-1}]	Assignment*
333	SO_3 rocking[5,6]
479	SO_3 bending[5]
501	SO_3 bending[5]
532	SO_3 bending[5,6]
767	C-S stretching[5,6]
897	S-OH stretching[5]
983	CH_3 rocking[5,6]
1122	SO_3 stretching[5]
1164	O-H bending[5]
1340	SO_3 stretching[5]
1418	CH_3 bending[5,6]
2945	Symmetric CH_3 stretching[5,6]
3033	Asymmetric CH_3 stretching[5,6]

*We find that there is evidence of hydrogen bonding within the molecules of methanesulfonic acid. Therefore, due to this intermolecular bonding, the three oxygen atoms become equivalent, which is why we refer the attribution to the SO_3 group instead of SO_2.

5. Electrochemical analysis

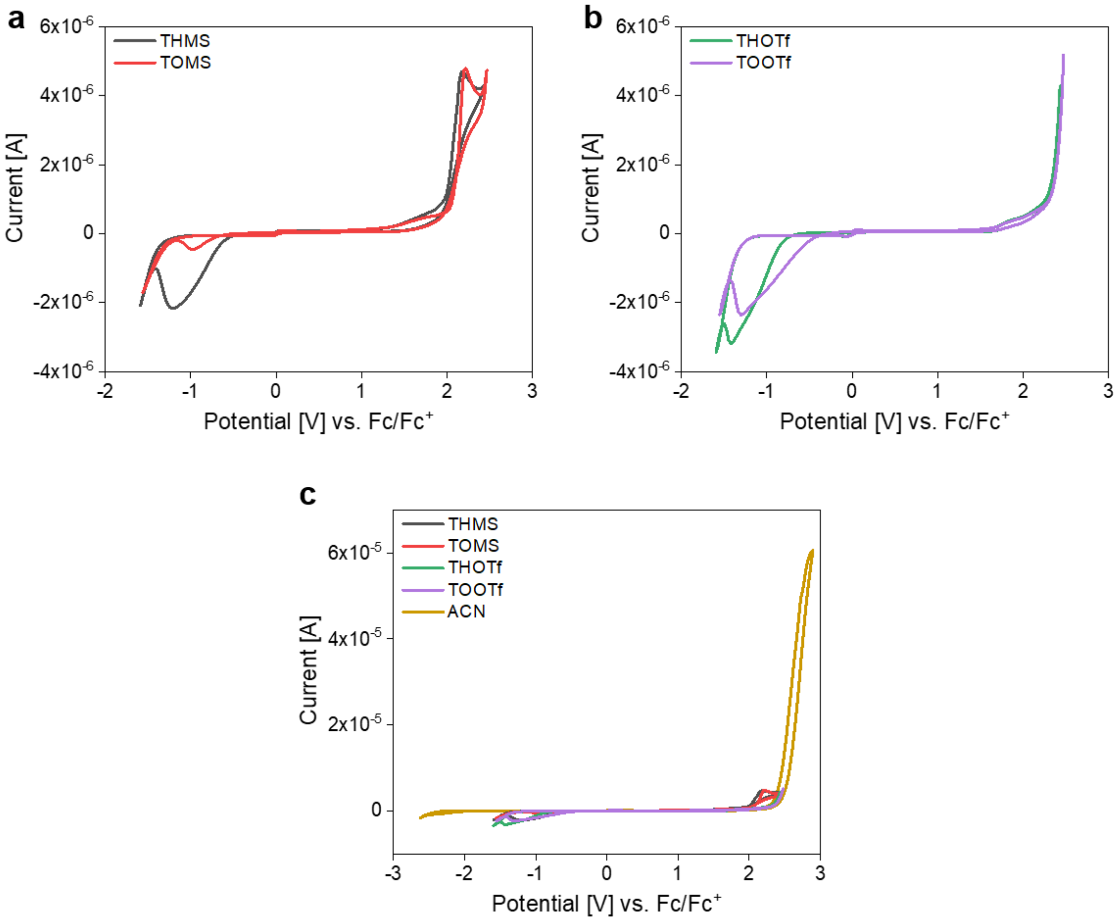

Figure S12: CV curves for a) protonated ILs, b) fluorinated ILs, c) all ILs and solvent (ACN).

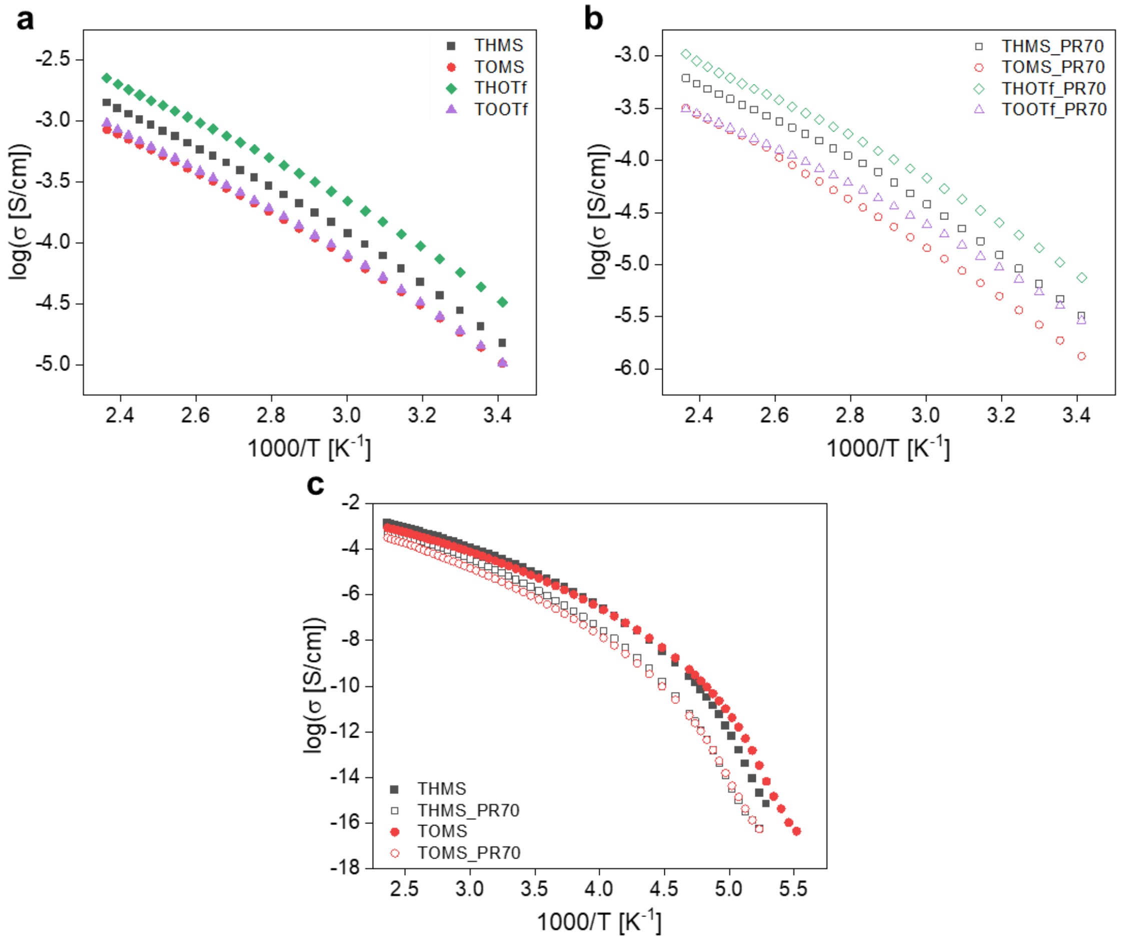

Figure S13: Ionic conductivities for a) pure ILs, b) IGs and c) protonated compounds until T_g.

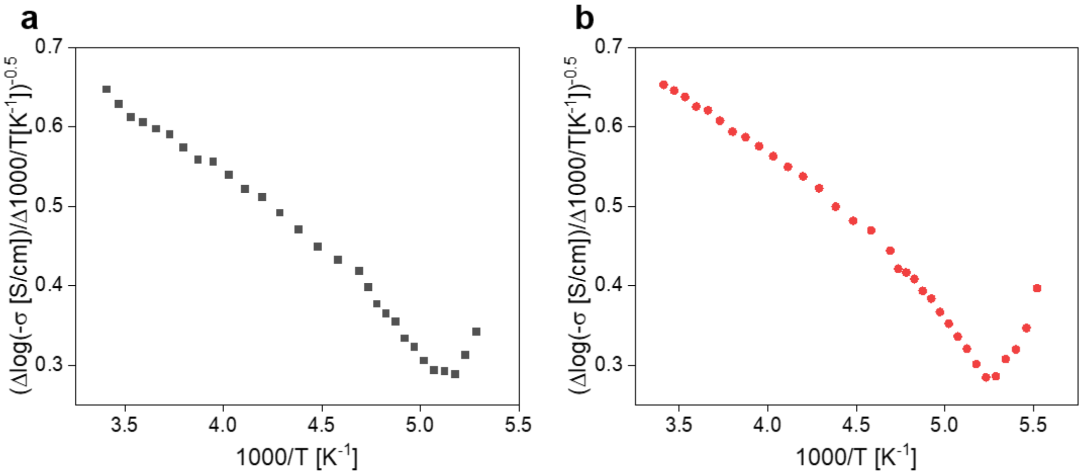

Figure S14: Representative Stickel plots for a) THMS and b) TOMS.

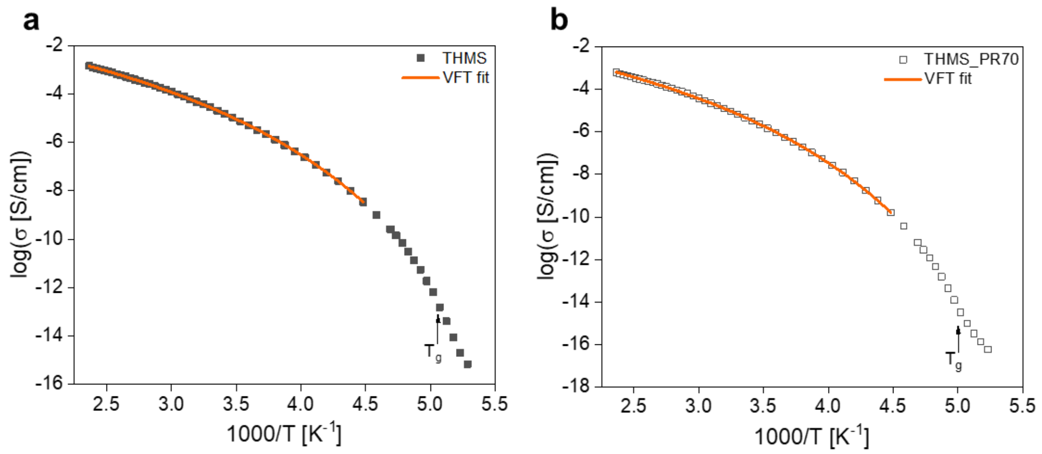

Figure S15: Representative VFT Fits for conductivities of a) THMS and b) THMS_PR70. The graphs were hereby fitted to the data points above 4.5 K^{-1} to stay well above the glass transition.

VFT fit: The graphs were hereby fitted to the data points above 4.5 K^{-1} (mesylate based compounds) and 3.4 K^{-1} (triflate based compounds) to stay above glass transition or crystallization processes.

6. IL densities

Table S3: Densities of pure ILs at 60 °C.

	Density ρ [g/mL]
**THMS**	0.9341
**TOMS**	0.9080
**THOTf**	1.0138
**TOOTf**	0.9738

References

(1) Larkin, P. *Infrared and Raman Spectroscopy: Principles and Spectral Interpretation*; Elsevier, **2018**.

(2) Cui, J. Synthesis of UV-Cured Hyperbranched Polyurethane Acrylate Coatings and Its Corrosion Resistance Revealed by Electrochemistry. *Int. J. Electrochem. Sci.* **2016**, 3727–3737.

(3) Mashouf, G.; Ebrahimi, M.; Bastani, S. UV curable urethane acrylate coatings formulation: experimental design approach. *Pigment & Resin Technology* **2014**, *43*, 61–68.

(4) Dapawan Kunwong, Natthawadee Sumanochitraporn, S. Kaewpirom. Curing behavior of a UV-curable coating based on urethane acrylate oligomer: the influence of reactive monomers. *Songklanakarin J. Sci. Technol.,* **2011**, 201–207.

(5) Zhong, L.; Parker, S. F. R. Soc. open sci. 2018, 5 (12), 181363.

(6) Capwell, R. J.; Rhee, K. H.; Seshadri, K. S. Spectrochimica Acta Part A: Molecular Spectroscopy. 1968, 24 (8), 955–958.

(7) Bodo, E.; Mangialardo, S.; Ramondo, F.; Ceccacci, F.; Postorino, P. J. Phys. Chem. B. 2012, 116 (47), 13878–13888.

(8) Paschoal, V. H.; Faria, L. F. O.; Ribeiro, M. C. C. Chem. Rev. 2017, 117 (10), 7053–7112.

(9) Pan, H.; Geysens, P.; Putzeys, T.; Gennaro, A.; Yi, Y.; Li, H.; Atkin, R.; Binnemans, K.; Luo, J.; Wübbenhorst, M. The Journal of Chemical Physics. 2020, 152 (23), 234504.

(10) Howell, N. K.; Arteaga, G.; Nakai, S.; Li-Chan, E. C. Y. J. Agric. Food Chem. 1999, 47 (3), 924–933.
